# Peroxisome proliferator activated receptor-γ agonist pioglitazone improves vascular and metabolic dysfunction in systemic lupus erythematosus

**DOI:** 10.1136/ard-2022-222658

**Published:** 2022-08-01

**Authors:** Sarfaraz Hasni, Yenealem Temesgen-Oyelakin, Michael Davis, Jun Chu, Elaine Poncio, Mohammad Naqi, Sarthak Gupta, Xinghao Wang, Christopher Oliveira, Dillon Claybaugh, Amit Dey, Shajia Lu, Philip Carlucci, Monica Purmalek, Zerai G Manna, Yinghui Shi, Isabel Ochoa-Navas, Jinguo Chen, Amrita Mukherjee, Kyu Lee Han, Foo Cheung, Galina Koroleva, Yasmine Belkaid, John S Tsang, Richard Apps, Donald E Thomas, Theo Heller, Massimo Gadina, Martin P Playford, Xiaobai Li, Nehal N Mehta, Mariana J Kaplan

**Affiliations:** 1 Lupus Clinical Trials Unit, Office of the Clinical Director, National Institute of Arthritis and Musculoskeletal and Skin Diseases, National Institutes of Health, Bethesda, Maryland, USA; 2 Systemic Autoimmunity Branch/NIAMS, National Institutes of Health, Bethesda, Maryland, USA; 3 Section of Inflammation and Cardiometabolic Diseases, National Heart, Lung, and Blood Institute, Bethesda, Maryland, USA; 4 Translational Immunology Section, NIH, National Institute of Arthritis and Musculoskeletal and Skin Diseases, Bethesda, Maryland, USA; 5 Office of the Clinical Director, NIH, National Institute of Arthritis and Musculoskeletal and Skin Diseases, Bethesda, Maryland, USA; 6 National Institutes of Health, Bethesda, Maryland, USA; 7 Metaorganism Immunity Section, Laboratory of Immune System Biology, National Institute of Allergy and Infectious Diseases, National Institutes of Health, Bethesda, Maryland, USA; 8 Multiscale Systems Biology Section, Laboratory of Immune System Biology, NIAID, National Institutes of Health, Bethesda, Maryland, USA; 9 NIH Center for Human Immunology, National Institutes of Health, Bethesda, Maryland, USA; 10 Arthritis and Pain Associates of PG County, Greenbelt, Maryland, USA; 11 NIDDK, National Institutes of Health, Bethesda, Maryland, USA; 12 NIAMS, National Institutes of Health, Bethesda, Maryland, USA; 13 National Heart Lung and Blood Institute, Bethesda, Maryland, USA; 14 Biostatistics and Clinical Epidemiology Service, National Institutes of Health Clinical Center, Bethesda, Maryland, USA

**Keywords:** systemic lupus erythematosus, cardiovascular diseases, lipids

## Abstract

**Objectives:**

Premature cardiovascular events in systemic lupus erythematosus (SLE) contribute to morbidity and mortality, with no effective preventive strategies described to date. Immune dysregulation and metabolic disturbances appear to play prominent roles in the induction of vascular disease in SLE. The peroxisome proliferator activated receptor-gamma agonist pioglitazone (PGZ suppresses vascular damage and immune dysregulation in murine lupus and improves endothelial dysfunction in other inflammatory diseases. We hypothesised that PGZ could improve vascular dysfunction and cardiometabolic parameters in SLE.

**Methods:**

Eighty SLE subjects with mild to severe disease activity were randomised to a sequence of PGZ followed by placebo for 3 months, or vice versa, in a double-blind, cross-over design with a 2-month wash-out period. Primary endpoints were parameters of endothelial function and arterial inflammation, measured by multimodal assessments. Additional outcome measures of disease activity, neutrophil dysregulation, metabolic disturbances and gene expression studies were performed.

**Results:**

Seventy-two subjects completed the study. PGZ was associated with a significant reduction in Cardio-Ankle Vascular Index (a measure of arterial stiffness) compared with placebo. Various metabolic parameters improved with PGZ, including insulin resistance and lipoprotein profiles. Circulating neutrophil extracellular trap levels also significantly decreased with PGZ compared with placebo. Most adverse events experienced while on PGZ were mild and resolved with reduction in PGZ dose.

**Conclusion:**

PGZ was well tolerated and induced significant improvement in vascular stiffness and cardiometabolic parameters in SLE. The results suggest that PGZ should be further explored as a modulator of cardiovascular disease risk in SLE.

**Trial registration number:**

NCT02338999.

**What is already known on this topic**

- Premature cardiovascular disease (CVD) in patients with systemic lupus erythematosus (SLE) is associated with significant morbidity and mortality. The underlying mechanisms of premature CVD in SLE are not well defined and no therapeutic agents have shown to significantly reduce CVD risk in SLE.

**What this study adds**

- The peroxisome proliferator-activated receptor-γ agonist pioglitazone is associated with improvement in vascular stiffness and various cardiometabolic parameters in SLE.

**How this study might affect research, practice or policy**

- These results have implications in using non-immunosuppressive therapy that could decrease CVD risk in patients with SLE.

## Introduction

Systemic lupus erythematosus (SLE) is a systemic autoimmune syndrome with heterogeneous clinical manifestations. While there has been substantial progress in treatment of SLE, this condition is still associated with significant morbidity and mortality, driven in part by premature cardiovascular disease (CVD).^1^ Depending on the study and outcome measure, the risk of CVD, especially in young women with SLE, can be as high as 50-fold when compared with matched controls.^2^ CVD driven by atherosclerosis develops or progresses in ~10% of SLE patients/year during short-term follow-up and is one of the most common causes of death.^3 4^ The traditional Framingham risk score cannot explain the CVD risk in SLE. Indeed, lupus is now recognised as an independent CVD risk factor.^5^

While the underlying mechanisms of premature CVD in SLE are not well defined, immune dysregulation coupled with cardiometabolic dysfunction are considered key drivers. This is exemplified by the characterisation of a pathophysiological alliance between type I Interferons (IFNs) and neutrophil dysregulation as inducers of vascular damage in SLE.^6–8^ In turn, aberrant formation of neutrophil extracellular traps (NETs) by SLE low-density granulocytes (LDGs) can oxidise lipoproteins and blunt the anti-atherogenic function of high-density lipoprotein (HDL). Furthermore, insulin resistance (IR) is highly prevalent in SLE, may be triggered in part by type I IFNs and other proinflammatory mediators and contribute to cardiometabolic dysfunction and atherosclerosis progression.^9 10^ Metabolic syndrome has also been associated with enhanced organ damage, vascular events and mortality in SLE.^11 12^ Recent evidence indicates that regulating innate immune pathways and inflammation in SLE can modulate various cardiometabolic parameters, including enhancing HDL’s cholesterol efflux capacity.^11 13^

In contrast, several attempts to modulate CV damage in SLE through the use of statins has given inconclusive or negative results.^14 15^ The use of some immunomodulators and immunosuppressives has been associated with a modest protective effect, but to date there are no therapeutic agents that have demonstrated to significantly reduce CVD risk in SLE.^16 17^

The thiazolidinediones (TZDs), including pioglitazone (PGZ), are a class of drugs approved for the treatment of patients with type 2 diabetes mellitus (DM). They belong to the family of drugs that activate the peroxisome proliferator-activated receptor-γ (PPAR-γ) and have been found to confer antiatherogenic and anti-inflammatory effects in diabetics and non-diabetic patient groups.^18^ In animal models of lupus, TZDs improved vascular damage, endothelial dysfunction and disease activity.^19–21^ Furthermore, PGZ improved vascular function and disease activity in rheumatoid arthritis.^22 23^

We hypothesised that PPAR-γ agonists may benefit SLE patients by suppressing inflammatory and immunologic pathways that promote CVD and internal organ damage. To test this hypothesis, we performed a double-blind, placebo-controlled, crossover study to test whether short term use of PGZ improves vascular function, vascular inflammation and various cardiometabolic parameters in SLE.

## Materials and methods

### Study design and subjects

The study design and conduct complied with relevant regulations regarding the use of human study participants and was conducted in accordance to the criteria set by the Declaration of Helsinki, as authorised by the NIH Office of Human Subject Research. After written informed consent and determination of eligibility, subjects were randomised to a sequence of PGZ followed by placebo (sequence AB), or placebo followed by PGZ (sequence BA) in a 1:1 allocation ratio, in a double-blind cross-over design. The starting dose of PGZ was 30 mg/day, which was titrated up to 45 mg after 1 week if tolerated. There was a 2-month wash-out period between the cross-over (online supplemental figure 1). Eighty SLE subjects that met the American College of Rheumatology Revised Criteria for the Classification of SLE and had mild to severe disease activity (Systemic Lupus Erythematosus Disease Activity Index 2000 (SLEDAI 2K) score between 4 and 20 or SLEDAI 2K ≥2 not considering anti-dsDNA or complement levels), and lack of A flares on the British Isles Lupus Activity Group (BILAG 2004) were enrolled in an outpatient clinical research setting.^24^ Eligible subjects were on stable doses of antimalarials and immunosuppressants (for 12 weeks prior to the screening visit) and/or oral glucocorticoids (for 2 weeks prior to the screening visit; prednisone or equivalent <20 mg/day). The primary outcome was change in the vascular function as measured by non-invasive vascular tests and the secondary outcome was decrease in SLE disease activity. The outcome variables were measured at baseline(day 1), and months 3, 5 and 8. The wash-out period was between months 3 and 5. SLE disease activity was determined using SLEDAI 2K, BILAG 2004, Physician Global Assessment (Likert scale 0–3) and patient-reported outcomes 36-item Short Form Survey (SF-36).^25–28^ Rate of adverse events (AEs, defined by the National Cancer Institute, Common Terminology Criteria for Adverse Events, V.4.0) was recorded at each visit.

10.1136/ard-2022-222658.supp1 Supplementary data

See online supplemental methods for assessments of vascular function, metabolic parameters, LDGs, NETs, transcriptional analysis, flow cytometry and statistical analysis.

## Results

### Characteristics of the cohort

**Figure 1:**
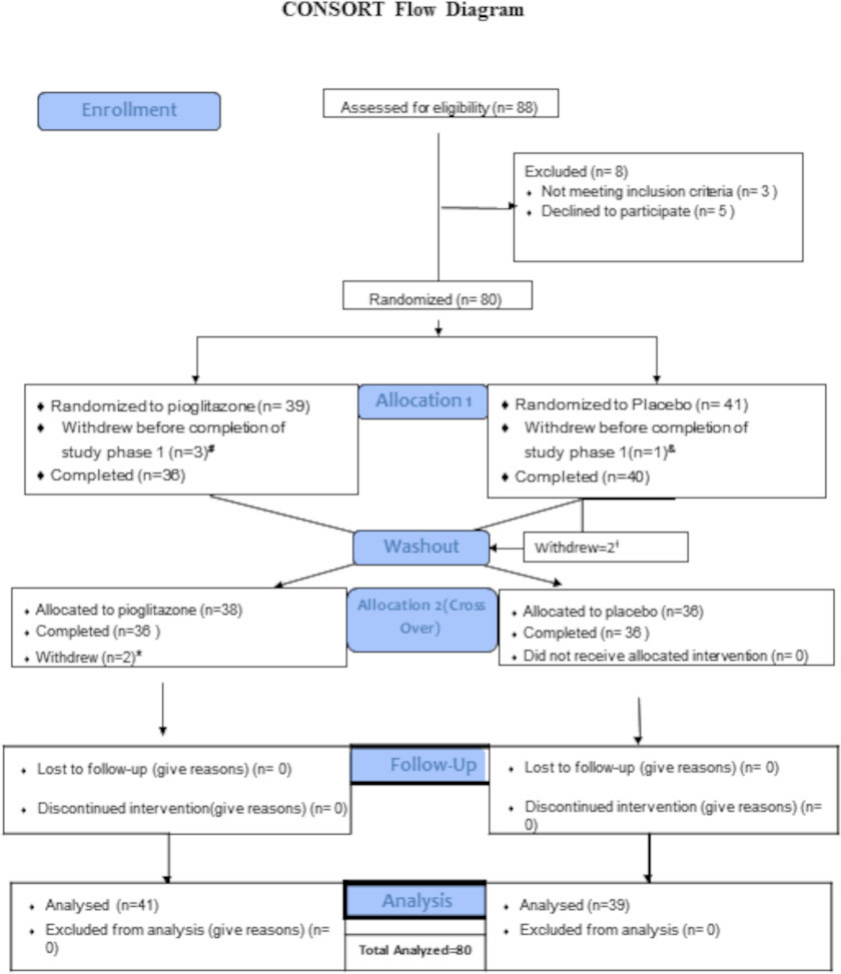
Consolidated Standards of Reporting Trials Flow Diagram. A total of 88 subjects were screened for the trial, with 80 subjects randomised to sequence AB (PGZ-wash-out-placebo N=39) or sequence BA (Placebo-wash-out-PGZ N=41). A total of 72 subjects completed all phases of the clinical trial. #Withdrew due to travel n=1; withdrew voluntarily due to AE (pruritus and increased urinary frequency) n=2; and lost to follow-up n=1. †Withdrawn due to SLE flare n=2. *Subject withdrew voluntarily due to weight gain n=2. AEs, adverse events; SLE, systemic lupus erythematosus.

Eighty subjects were randomised and took at least one dose of the drug and 72 completed all phases of the study. Four subjects withdrew due to AEs (pruritus, weight gain, polyuria), two due to SLE flare, one each due to travel constraints and lost to follow-up (figure 1). Baseline demographics were similar in both sequences (PGZ-Placebo (AB) and Placebo-PGZ (BA); table 1). Consistent with SLE demographics, 87.5% were females, with mean±SD age 45.7±12.1 years and mild-to-moderate disease activity (SLEDAI 2K : 5.1±2.88).

**Table 1:** Baseline characteristics of study subjects

	Pioglitazone-placebo group=sequence AB	Placebo-Pioglitazone group=sequence BA	Total
	N=39	N=41	N=80
Race/ethnicity: N (%)			
Hispanic	16 (41)	16 (39)	32 (40)
Caucasian	9 (23)	9 (22)	18 (22.5)
African American	9 (23)	8 (19.5)	17 (21.25)
Asian	4 (10)	5 (12)	9 (11.25)
Multi	0 (0)	1 (2)	1 (1.25)
Unknown	1 (2.5)	2 (5)	3 (3.75)
Female: N (%)	33 (84.6)	37 (90.2)	70 (87.5)
Male: N (%)	6 (15.4)	4 (9.8)	10 (12.5)
Age (years) mean (SD)	46.03 (13.79)	45.32 (10.41)	45.66 (12.1)
Disease duration (years) mean (SD)	13.59 (11.64)	12.59 (10.46)	13.08 (10.99)
BMI mean (SD)	28.52 (5.76)	30.49 (7.5)	29.53 (6.74)
SLEDAI 2K mean (SD)	5.13 (2.75)	5.07 (3.04)	5.1 (2.88)

Descriptive statistics were used to characterise patients for continuous variables using mean and SD. For categorical variables frequencies and (%) percentages were used.

BMI, body mass index; SLEDAI 2K, Systemic Lupus Erythematosus Disease Activity Index 2000.

### Pioglitazone improves arterial stiffness

**Figure 2:**
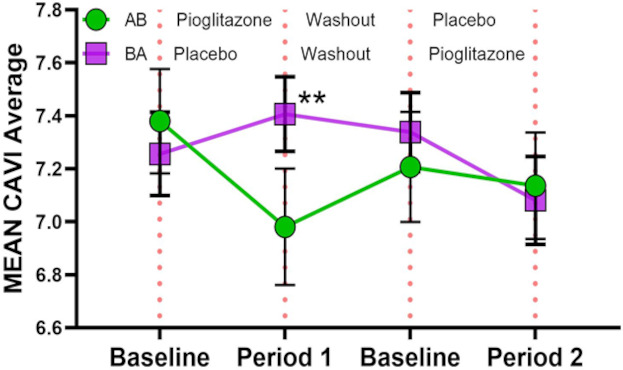
PGZ improves vascular stiffness in SLE. Mean Cardio-Ankle Vascular Index (CAVI) average of right and left side in subjects randomised to sequence AB (N=39; PGZ-wash-out-placebo) and sequence BA (N=41; placebo-wash-out-PGZ). The CAVI values decreased by 0.32 points (95% CI 0.10 to 0.54, p=0.005) in the pioglitazone group compared with the placebo. All data presented as mean+SD. **p≤0.01.

PGZ use was associated with a significant decrease in arterial stiffness, as determined by Cardio-Ankle Vascular Index (CAVI) (0.37±0.9 in period 1 and −0.27±0.56 period 2 when PGZ was given vs 0.11±0.65 in period 1 and −0.07±0.66 in period 2 when placebo was given(figure 2). CAVI values decreased by 0.32 points more (95% CI -o.54,-0.10; p=0.005, table 2) in the PGZ group compared with placebo. CAVI values reverted to baseline during the wash-out period and while subjects were on placebo. Other measures of vascular stiffness (PWV and RHI) did not display significant improvement with PGZ (p=0.37 and 0.91, respectively, table 2).

**Table 2:** Summary of vascular and metabolic variables by sequence and period

Variable (mean±SD)	Sequence AB (pioglitazone/placebo) N=39				Sequence BA (placebo/pioglitazone) N=41				Treatment effect*	
	Period 1 (pioglitazone)		Period 2 (placebo)		Period 1 (placebo)		Period 2 (pioglitazone)			
	Baseline	Change	Baseline	Change	Baseline	Change	Baseline	Change	Estimate (95% CI)	P value
CAVI average	7.38±1.23	−0.37±0.90	7.21±1.25	−0.07±0.66	7.26±1.01	0.11±0.65	7.34±0.92	−0.27±0.56	−0.32 (-0.54, - 0.10)	0.005
Log-transformed RHI	0.72±0.37	0.07±0.35	0.71±0.29	0.02±0.36	0.65±0.36	0.02±0.48	0.64±0.54	−0.05±0.35	−0.007 (-0.123 0.110)	0.91
PWV m/s	6.59±1.82	−0.31±2.06	7.15±1.22	−0.25±0.94	6.73±1.52	0.03±2.03	7.01±2.44	−0.31±1.86	−0.18 (-0.57,0.21)	0.37
Augmentation Index	23.59±16.33	−2.25±11.84	26.28±15.90	−1.75±10.01	26.24±10.9	−0.43±10.32	23.61±9.64	0.47±12.33	−1.36 (-4.11, 1.39)	0.33
Aortic Arch TBR*†	1.47±0.16	0.09±0.12			1.53±0.19	−0.05±0.27			0.01 (-0.16, 0.18)	0.84
Global TBR*†	1.68±0.14	0.03±0.20			1.66±0.19	0.04±0.30			0.09 (-0.04, 0.23)	0.17
Cholesterol	174.97±30.04	0.06±14.74	177.42±31.48	−2.44±15.83	169.71±30.18	1.35±21.73	171.58±36.69	−0.44±21.39	0.38 (-5.17, 5.92)	0.89
LDL mg/dL	90.9±29.38	0.14±14	93.58±28.43	−0.47±13.05	88.29±26.53	−0.88±18.64	93.32±31.95	−6.19±24.33	−1.65 (-6.38,3.09)	0.91
Triglycerides mg/dL	101.67±40.52	−20.94±39.57	97.19±44.85	−2.72±36.24	110.35±52.1	6.54±47.97	109.97±67.84	−14.47±45.33	−18.09 (-30.52, -5.67)	0.005
HDL mg/dL	63.79±20.37	4.14±12.29	64.33±18.29	−1.36±7.91	59±20.91	1.28±10.42	59.34±22.93	5.42±11.53	4.72 (1.27, 8.18)	0.008
HDL particle no mcmol/L	31.2±6.6	−1.58±3.83	31.13±7.41	0.18±2.62	31.36±6.51	0.11±3.45	31.61±6.65	−1.76±4.23	−1.84 (-2.90,- 0.78)	0.0009
HDL size nm	9.79±0.66	0.27±0.42	9.84±0.63	−0.04±0.27	9.58±0.63	0.02±0.35	9.6±0.65	0.29±0.37	0.28 (0.18, 0.39)	<0.0001
LDL particle no nmol/L	958.1±396.6	−83.4±234.1	977.3±401.5	17.4±192.2	1037.5±387.4	−7.9±201.6	1032.1±411.5	−140.6±268.6	−117.1 (-183.3, -51.0)	0.0006
LDL size nm	20.86±0.52	0.49±0.66	21.14±0.57	−0.15±0.42	20.85±0.68	−0.11±0.48	20.87±0.63	0.43±0.74	0.51 (0.38, 0.71)	<0.0001
Cholesterol efflux value	0.92±0.17	0.03±0.16	0.88±0.19	0.04±0.18	0.88±0.19	0.01±0.17	0.89±0.2	0.07±0.16	0.03 (-0.02, 0.08)	0.28
Glucose mg/dL	89.74±14.74	−3.69±16.08	89.92±10.26	−1.72±6.44	88.66±9.07	−0.65±6.85	88.92±9.99	−3.17±9.15	−1.91 (-4.39, 0.58)	0.13
Insulin Pmol/L	17.26±10.66	−4.02±9.9	18.98±19.26	−2.85±9.02	17.63±11.71	1.18±6.21	17.74±10.19	−5.46±8.59	−3.77 (-6.22,- 1.31)	0.0031
Homa2-IR	2.18±1.34	−0.51±1.28	2.36±2.15	−0.33±0.98	2.22±1.41	0.14±0.73	2.24±1.25	−0.69±1.08	−0.23 (-0.35,- 0.11)	0.0003

Data are mean±SD. Change is defined as the post baseline value minus the baseline value during the period: that is, M3 – D1 for period 1, M8 – M5 for period 2.

*Linear mixed effects models were used to calculate the estimated treatment effect (the treatment group difference in the change score between pioglitazone and the placebo), its 95% CI and the p value.

†These measures are based on 18F-FDG/PET CT scans. For these two variables measured in period 1 only, the treatment effect and the p value are calculated based on analysis of covariance.

CAVI, Cardio-Ankle Vascular Index; ^18^F-FDG/PET CT scan, 18fluoro-D-glucose positron emission tomography integrated with CT; HDL, high-density lipoprotein; HOMA2IR, Homoeostasis Model Assessment of Insulin Resistance; LDL, low-density lipoprotein; PWV, pulse wave velocity; RHI, Reactive Hyperaemia Index; TBR, target/background ratio.

18fluoro-D-glucose positron emission tomography integrated with CT scans were performed on 30 subjects who consented to the procedure and analysis did not reveal significant changes in vascular inflammation after 3 months of PGZ (Aortic arch TBR p=0.84, Global TBR p=0.17). Overall, PGZ use for 3 months in mild-to-moderate SLE resulted in significant improvements in arterial stiffness, as assessed by CAVI.

### Pioglitazone improves cardiometabolic parameters

**Figure 3:**
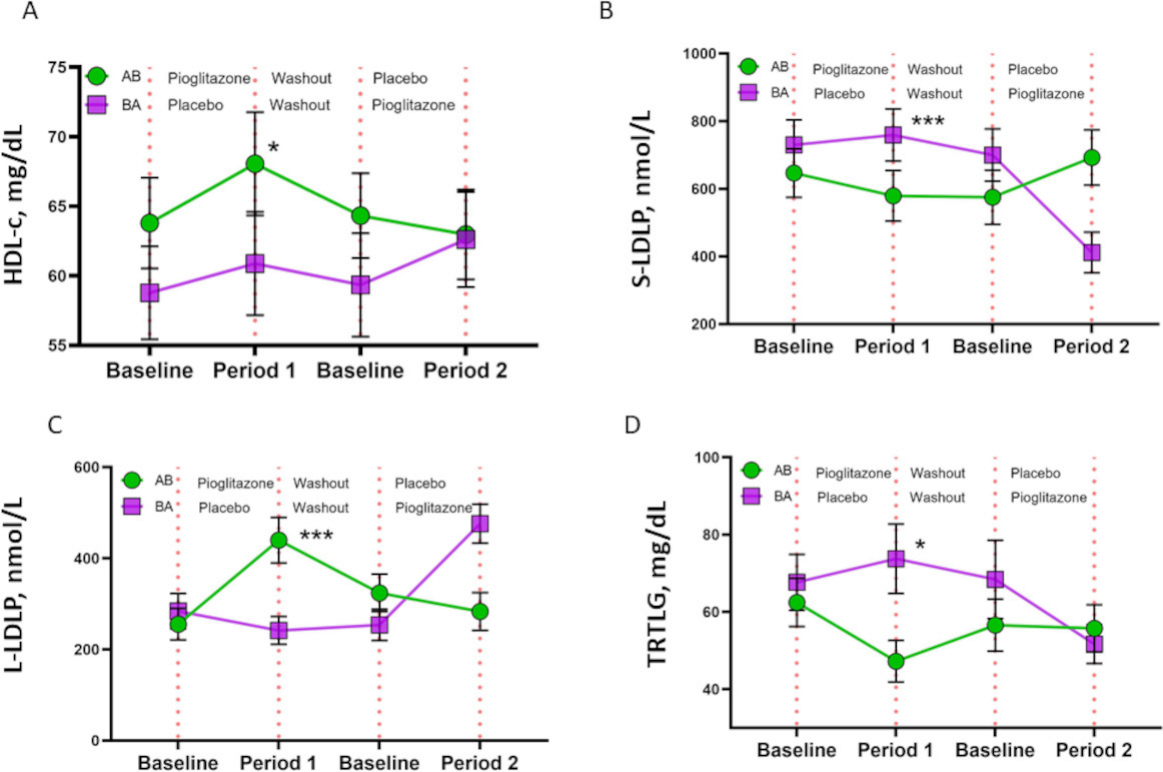
PGZ improves lipoprotein profiles in SLE. (A) Mean circulating HDL in subjects randomised to sequence AB (N=39; PGZ-wash-out-placebo) and sequence BA (N=41; placebo-wash-out-PGZ). The serum HDL levels increased by 4.72 mg/dL (95% CI: (1.27 to 8.18) with PGZ compared with placebo, with return to baseline by the end of wash-out period; p=0.008. (B) Mean circulating small LDL particles (s-LDLP) in subjects randomised to sequence AB (N=39; PGZ-wash-out-placebo) and sequence BA (N=41; placebo-wash-out-PGZ). The serum s-LDLP levels redcued by −254.76 (95% CI −354.13 to −155.39) with PGZ compared with placebo; p<0.0001. (C) Mean circulating large LDL particles (l-LDLP) in subjects randomised to sequence AB (N=39; PGZ-wash-out-placebo) and sequence BA (N=41; placebo-wash-out-PGZ). The serum l-LDLP levels increased by 236.68 (95% CI (182.47 to 290.89) with PGZ compared with placebo; p<0.0001. (D) Mean circulating triglyceride-rich lipoproteins (TRLs) in subjects randomised to sequence AB (N=39; PGZ-wash-out-placebo) and sequence BA (N=41; placebo-wash-out-PGZ). The serum TRL levels redcued by −16.03 (95% CI (−26.98 to −5.07) with PGZ compared with placebo; p=0.01. All data presented as mean+SD. *P≤0.05; ***p≤0.001. HDL, high-density lipoprotein; PGZ, pioglitazone; SLE, systemic lupus erythematosus.

There were improvements in serum lipoproteins and IR with the PGZ use. Serum HDL levels increased with PGZ (4.14±12.29 in period 1; 5.42±11.53 in period 2) compared with placebo (1.28±10.42 in period 1; −1.36±7.91 in period 2). Overall, the increase was 4.72 mg/dL more (95% CI 1.27 to 8.18), p=0.008) in PGZ than in placebo (figure 3A). Similarly, HDL particle size increased by 0.28 (95% CI 0.18 to 0.39, p<0.0001) and the particle number decreased by −1.84 (95% CI −2.90 to −0.78, p=0.0009). Conversely, there was an increase in low-density lipoprotein (LDL) particle size by 0.51 (95% CI 0.38 to 0.71, p<0.0001) and decrease in LDL particle number by −117.1 (95% CI −183.3 to −51.0, p=0.0006) with PGZ(table 2). The concentration of small LDL particles (s-LDLP) decreased by −254.76 (95% CI −354.13 to −155.39, p<0.0001) whereas the concentration of large LDL particles increased by 236.68 (95% CI 182.47 to 290.89, p<0.0001) on PGZ treatment (figure 3B, C). Serum triglyceride levels decreased with PGZ by −20.94±39.57 mg/dL during period 1 and by −14.47±45.33 mg/dL during period 2, with an overall reduction by −18.09 mg/dL (95% CI −30.52 to –5.67, p=0.005) with PGZ use compared with placebo. We also noted a PGZ-mediated decrease in triglyceride-rich lipoproteins (TRLs) of −16.03 (95% CI −26.98 to −5.07, p=0.01), another subset of lipoproteins considered to be causal for atherosclerotic CVD (figure 3D). There were no significant changes in cholesterol efflux capacity with PGZ use (table 2).

**Figure 4:**
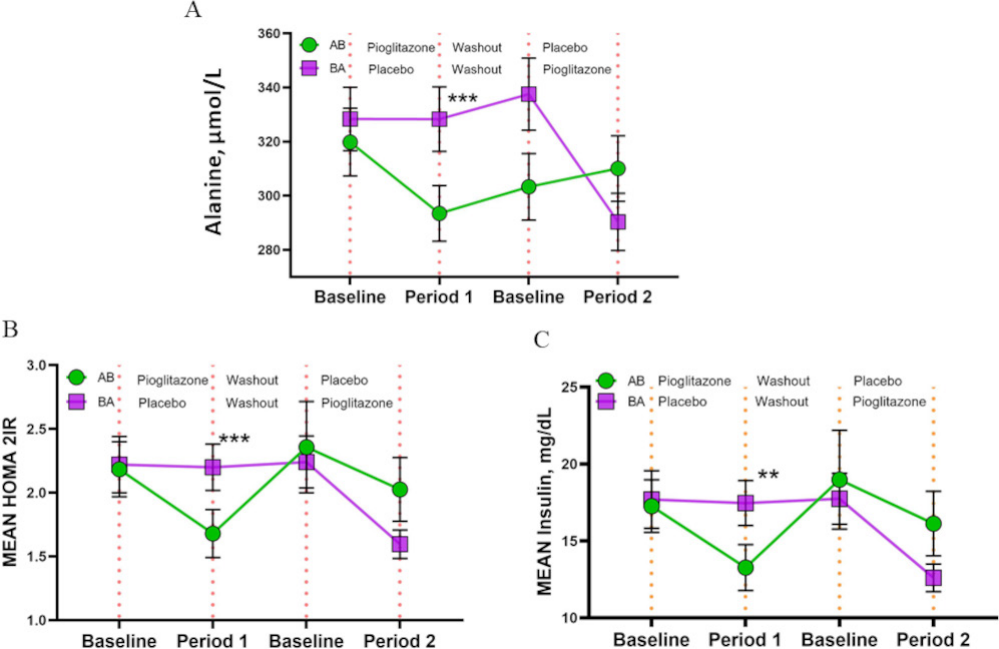
PGZ reduces serum alanine and improves insulin resistance in SLE. (A) Mean circulating serum alanine levels in subjects randomised to sequence AB (N=39; PGZ-wash-out-placebo) and sequence BA (N=41; placebo-wash-out-PGZ). The serum alanine levels redcued by −33.35 (95% CI (−51.29 to −15.4), with PGZ compared with placebo; p=0.0004. (B) Mean homoeostasis model assessment of IR (HOMA2-IR) in subjects randomised to sequence AB (N=39; PGZ-wash-out-placebo) and sequence BA (N=41; placebo-wash-out-PGZ). HOMA IR levels decreased by −0.23 (95% CI (−0.35 to −0.1), p=0.0003), respectively PGZ a compared with placebo. (C) Mean serum insulin levels in subjects randomised to sequence AB (N=39; PGZ-wash-out-placebo) and sequence BA (N=41; placebo-wash-out-PGZ). Overall, the serum insulin levels decresased by-3.77 (95% CI (−6.22 to –1.31), p=0.003) with the use of pioglitazone as compared with placebo. All data presented as mean+SD; **p≤0.01; ***p≤0.001. PGZ, pioglitazone; SLE, systemic lupus erythematosus.

There was reduction in circulating alanine with the PGZ use by −33.35 (95% CI −51.29 to −15.4, p=0.0004) (figure 4A). There were 37 subjects (51.4%) with evidence of IR (Homoeostasis Model Assessment of IR (HOMA IR cut-off >1.9) at the beginning of the trial. With PGZ use, 18 (48.6%) of these subjects had normalisation in HOMA IR. Baseline insulin and HOMA IR levels were 16.86+9.65 mcU/mL and 2.13±1.21, respectively. While on PGZ, serum insulin and HOMA IR levels decreased by −4.02±9.9 and −0.51±1.28 during period 1; by −5.46±8.59 and −0.69±1.08 during period 2 (p=0.003 and p=0.0003), respectively; figure 4B, C). Overall, serum insulin and HOMA IR levels decreased by-3.77 (95% CI −6.22 to –1.3, p=0.003) and −0.23 (95% CI −0.35 to −0.11, p=0.0003), respectively, with PGZ use compared with placebo. Serum glucose was not significantly modified with PGZ use (p=0.13). All metabolic parameters returned to baseline values during wash-out and placebo phases. Overall, short-term use of PGZ resulted in significant improvements in lipoprotein profiles, a significant shift in LDL particle number from a high to lower pro-atherogenic form and improved IR in mild-to-moderate SLE.

### Pioglitazone does not modify interferon-stimulating genes but decreases NET levels

PGZ use did not alter interferon-stimulating genes (ISGs), as assessed by Nanostring (online supplemental figure 2). While PGZ use was not associated with changes in LDG levels, it was associated with lower levels of circulating NET remnants (p=0.026; online supplemental figure 3). There were no significant changes in soluble markers of endothelial cell activation (sL-selectin, sICAM-1 and sVCAM-1) with the use of PGZ. As the targeted analysis of inflammation-related genes showed no effect when subjects were treated with PGZ, we performed unbiased screening to detect potentially other effects of this drug on immune phenotype. Whole blood transcriptomic analysis and high parameter cytometry phenotyping of peripheral blood mononuclear cells was done on a subset of patients who had demonstrated the greatest improvements in CAVI when treated with PGZ, but no changes in either could be attributed to PGZ (online supplemental material). Overall, short-term use of PGZ did not modify ISGs and other immune related parameters but did lower the levels of circulating NETs.

### Safety and tolerability

PGZ was well tolerated and did not affect disease activity in mild-to-moderate SLE. SLE disease activity, as measured by SLEDAI-2K, remained stable during the trial (online supplemental table 3). Two subjects developed moderate lupus flares during the wash-out period and withdrew from trial, as escalation of immunosuppressive therapy was not allowed while in the study. There was an increase in serum C4 levels associated with PGZ use (p=0.04) while the rest of the serological parameters (C3 and anti-ds-DNA antibody) did not show significant changes (online supplemental table 3). Self-reported disease outcomes, as measured by SF-36, showed a trend towards improvement with PGZ that was not statistically significant (p=0.08).

There were 249 AEs recorded during the study, with no significant difference in overall AEs between the two groups (52.6% of AEs on PGZ and 47.4% of AEs on placebo). The majority of AEs (67.5%) were mild and resolved without any intervention; there was one urinary tract infection requiring hospitalisation in a subject while on placebo. Overall, there were more infections while subjects were on placebo (table 3). No deaths occurred during the study (online supplemental table 4). Weight gain, fluid retention and mild transaminitis were noted in nine subjects on titrating up PGZ dose to 45 mg /day and these events either self-resolved or resolved after dose reduction to 30 mg/day. There were no cases of new onset hematuria, bladder cancer, congestive heart failure or fragility fractures during the study. Most laboratory tests remained stable, with changes that were not clinically significant but with some that were statistically significant and most likely due to volume overload (online supplemental table 5). Overall, PGZuse was well tolerated in SLE and was associated with an improvement in C4 complement proteins but no significant changes in disease activity in patients with mild-to-moderate SLE.

**Table 3:** Adverse events (AE) by body system and treatment

Body system preferred term severity	Pioglitazone (N=77)	Placebo (N=77)
	n (%)	n (%)
Blood and lymphatic system disorders	5 (6.5)	1 (1.3)
Cardiac disorders	4 (5.2)	1 (1.3)
Eye disorders	1 (1.3)	3 (3.9)
Gastrointestinal disorders	15 (19.5)	14 (18.2)
General disorders	8 (10.4)	5 (6.5)
Immune system disorders	0 (0.0)	1 (1.3)
Infections and infestations	15 (19.5)	27 (35.1)
Injury poisoning and procedural complications	0 (0.0)	1 (1.3)
Investigations*	15 (19.5)	7 (9.1)
Metabolism and nutrition disorders	4 (5.2)	1 (1.3)
Musculoskeletal and connective tissue disorders	4 (5.2)	4 (5.2)
Nervous system disorders	16 (20.8)	12 (15.6)
Psychiatric disorders	2 (2.6)	2 (2.6)
Renal and urinary disorders	5 (6.5)	2 (2.6)
Reproductive system and breast disorders	0 (0.0)	2 (2.6)
Respiratory thoracic and mediastinal disorders	6 (7.8)	9 (11.7)
Skin and subcutaneous tissue disorders	3 (3.9)	2 (2.6)
Surgical and medical procedures	1 (1.3)	0 (0.0)
Vascular disorders	1 (1.3)	2 (2.6)

n=number of subjects who had specific AE at least once; % of total number of subjects.

A total of 13 SAE were observed in 10 subjects. Eight SAEs while on placebo and five while on pioglitazone. All SAEs were followed until resolved.

*Abnormal lab values.

SAE, serous adverse event.

## Discussion

CVD due to accelerated atherosclerosis is a significant contributor of morbidity and mortality in SLE and the effect of drugs currently used to treat SLE on improving cardiometabolic parameters and CV risk in SLE has not been systematically demonstrated. Antimalarials may display a mild vasculo-protective role due to pleiotropic effects on the immune system^16^ while some immunosuppressive roles may have mild protective effects that remain to be demonstrated in larger patient populations.^17^ As such, finding interventions that can modulate lupus vasculopathy, modify cardiometabolic risk and not further immunosuppress these patients is an area of great need in this disease. In the current study, we showed that PGZ, when used in non-diabetic patients with mild to moderate SLE, improves arterial stiffness and various metabolic parameters associated with increased CVD risk. The results of the study support previous observations that TZDs have immunomodulatory and vasculo-protective roles in murine models of lupus and in patients with RA.^20–22^

Arterial stiffness, as measured by CAVI, was the main vascular parameter that improved during PGZ use. CAVI measures the stiffness of the arterial tree from the origin of the aorta to the ankle and has been shown to be an independent CVD risk factor and a putative surrogate end-point marker for vascular disease risk.^29^ SLE patients have higher incidence of abnormal CAVI, and this may contribute to their increased CVD risk.^30^ Supporting previous studies, the baseline CAVI values in SLE subjects in this study were significantly higher than the reference value for age and gender-matched healthy volunteers,^31^ indicating that SLE subjects with mild to moderate disease display significant arterial stiffness that improves with short-term use of PGZ. In contrast, other vascular function measurements did not significantly change with PGZ. While the implications of these discrepancies using the different vascular function assessments remains to be determined, these results support the need for multimodal measurements of vascular function to better understand how different vascular territories are affected in SLE. Vascular inflammation that was measured in a subset of the subjects enrolled in the study did not show significant changes after 3 months of PGZ. The reasons for this lack of response may be related to the short duration of drug exposure that may not had been sufficiently long to lead to changes in inflammation of the vessel wall, in contrast to the metabolic effects that occurred within the timeframe of the study that could have benefited vascular function. In contrast, changes in systemic immune parameters that could have contributed to alter vascular wall inflammation were not modified during the trial, with the exception of NET levels. Another possibility for the lack of detected effect on arterial wall inflammation could have been that the FDG-PET-CT was performed only in a subset of patients in the study and the sample size may not have allowed to detect these differences. It is possible that the impact of PGZ on vascular function in SLE is not related to immune regulation but, rather, to modifications of metabolic parameters known to have significant impact on vascular disease.

In previous studies in non-lupus populations, PGZ was effective in primary and secondary CVD prevention and in modulating renal AEs in individuals with or at high risk to develop type 2 DM.^32^ SLE patients have well described abnormalities in IR and lipoprotein profiles, with proatherogenic consequences.^33^ In a previous small clinical trial, PGZ administration over 3 months led to improvements in HDL levels, IR and HDL size, while decreasing markers of inflammation such as C reactive protein and serum amyloid A.^34^ In the current study, PGZ use was associated with increases in HDL, HDL particle size and number, reduced triglycerides and TRLs, a switch from s-LDLP to less atherogenic larger ones, reduced alanine and improved IR. The decrease in circulating alanine levels with PGZ treatment may be due to previously reported effects of PPAR agonism on Alanine Aminotransferase activity, which converts alanine to pyruvate and glutamate.^35^ The clinical significance of this finding is a subject for future investigation. As expected, the improvement in HOMA-IR was due to reduced serum insulin levels without a drop in serum glucose, which is important from a safety perspective in these non-diabetic patients. While cholesterol efflux capacity was not altered in this study, the changes in lipoprotein profile may confer additional antiatherogenic effects beyond this measurement. This remains to be determined in the future studies.

There are concerns with the use of pioglitazone in diabetics, such as fluid retention, increased risk of fracture and bladder cancer.^36–38^ In SLE, short term PGZ use was well tolerated and the side effects were consistent with what has been described in the literature, including peripheral oedema and mild transaminitis in a small proportion of patients. There were no fractures, new onset of hematuria or bladder cancer during the study. However, whether longer exposure to this drug in SLE can promote these complications remains to be determined.

The subjects enrolled in this study had overall low SLE disease activity at enrollment. As such, the probability to observe any significant improvements in disease activity would be limited and the study was not designed or powered to assess the role of this drug in disease activity. Subjects were kept on standard of care and any escalation in dose or addition of new medication for SLE would result in withdrawal from the study, which further precluded establishing immunomodulatory roles of the drug in this disease. Of note, C4 levels increased significantly while on PGZ, indicating some potential role in normalising biomarkers of disease activity in SLE. This should be explored in future studies. The lack of significant changes in disease activity was paired to the observation that use of this drug for a limited period of time did not result in significant modulation of the type I IFN response, or changes in cytokine levels. Therefore, it is possible that the favourable effect on arterial stiffness promoted by this drug was secondary to the effects on lipoprotein parameters and IR. However, NET levels decreased while SLE patients were on PGZ, indicating a putative immunomodulatory effect on dysregulated neutrophil biology previously described in SLE. Whether this decrease in NETs contributed to improving vascular stiffness remains to be determined in follow-up studies, given that NETs have been found to be linked to vascular disease in lupus and other chronic inflammatory conditions.

Limitations of this study come from the relatively short duration of the study and the inclusion of only of mild-to-moderate SLE patients, which precluded our ability to further investigate how the drug modulated disease activity and more severe vascular disease. As mentioned above, the ability to check vascular inflammation only in a subset of the patients, limited the ability to evaluate the role of this drug in this specific parameter.

In summary, PGZ was well tolerated during short-term use in SLE, and was associated with significant improvements in arterial stiffness and various cardiometabolic parameters considered to be CVD risk factors. Exploring whether PPAR-γ modulation, with PGZ or other newer generation drugs, can mitigate organ damage and disease manifestations in SLE while maintaining an adequate safety profile should be explored in future studies.

## Data Availability

Data are available on reasonable request. All deidentified individual subject and clinical trials data will be made available after executing material transfer agreements as applicable under the laws of US Federal Government.
