# Supplementary material for: Peroxisome proliferator activated receptor-γ agonist pioglitazone improves vascular and metabolic dysfunction in systemic lupus erythematosus
Source: Ann Rheum Dis. 2022 Aug 1;81(11):1576–84. doi: 10.1136/ard-2022-222658 (PMC9606512; doi:10.1136/ard-2022-222658)
Supplement: Supplementary data [file ard-2022-222658supp001.pdf]

BMJ Publishing Group Limited (BMJ) disclaims all liability and responsibility arising from any reliance placed on this supplemental material which has been supplied by the author(s)

Table of Contents

Supplemental methods: 2
- Measurements of cardiovascular risk factors and vascular function. 2
- Non -invasive vascular function studies: 2
- Quantification of LDGs: 4
- NETs ELISA: 4
- ELISAs for soluble markers of endothelial cell activation: 4
- ^{18}F-FDG–PET/CT: 5
- RNA Isolation and IFN Gene Signature (ISG) quantification by Nanostring: 5
- High dimensional transcriptional, and flow cytometry analysis. 6
  - Sample collection and selection for high dimensional analysis: 6
  - Transcriptomics: 6
  - Flow Cytometry: 7
  - Data analysis: 7
- Statistical Analysis: 8

Supplemental Results 9
- Supplemental Figures: 11
- Supplemental Tables: 14
- References: 38

Hasni S, *et al. Ann Rheum Dis* 2022; 81:1576–1584. doi: 10.1136/ard-2022-222658

Supplemental methods:

Measurements of cardiovascular risk factors and vascular function.

The homeostasis model assessment of insulin resistance (HOMA-IR) index = fasting glucose (mmol/l) × fasting insulin (μU/ml)/22.5) was used to estimate insulin resistance (1). Overnight fasting lipid profiles were obtained at the NIH Clinical Center Central Laboratories. Lipoprotein particle concentration and diameters were measured using automated Nuclear Magnetic Resonance Spectroscopy (NMR) using the LP4.20 algorithm. The HDL cholesterol efflux capacity was measured using published methods(2). Briefly, $3x10^5$ J774 cells/well were seeded in 24-well plate and radiolabeled with 2 μCi of 3H-cholesterol/mL in RPMI-1640 media containing 1% FBS for 24-hours. Cells were incubated for 16-hours in RPMI/2% BSA in the presence or absence of 0.3 mmol/L 8-(4-chlorophenylthio)-cAMP to upregulate ATP-binding cassette transporter A1. This was followed by addition of 2.8% apoB-depleted plasma to the efflux medium for 4 hours. A liquid scintillation counter was used to quantify the efflux of radioactive cholesterol from cells using the formula: (μCi of 3H-cholesterol in media containing 2.8% apoB-depleted subject plasma-μCi of 3H-cholesterol in plasma-free media /μCi of 3H-cholesterol in media containing 2.8% apoB-depleted pooled control plasma-μCi of 3H-cholesterol in pooled control plasma-free media). Pooled plasma was obtained from five healthy adult volunteers. All assays were performed in duplicate. LCAT concentration was quantified by ELISA (BioVendor; Ashville, NC).

Non -invasive vascular function studies:

These studies included the cardio-ankle vascular index (CAVI), peripheral arterial tonometry (PAT; reactive hyperemia index (RHI), and pulse wave velocity (PWV) (3). Subjects were asked to fast for at least 6 hours prior to these tests and to refrain from smoking or drinking caffeinated beverages for 24 hours prior to the studies. Subjects were asked to hold vasodilators,

anti-hypertensives and statins on the morning of the test. During testing, subjects were placed in a temperature-controlled quiet room in the supine position.

1) CAVI**.** CAVI was measured using VaSera-1500A (Fukuda Denshi Co. Redmond, WA). After placing blood pressure (BP) cuffs around both arms and ankles and attaching electrocardiogram electrodes to the upper arms, a microphone was placed on the sternal angle to record heart sounds. Measurements were automatically calculated using the VaSera VS-1000 software. The principle underlying CAVI has been discussed previously.(4)

2) PAT. Microvascular endothelial function was evaluated using PAT with an EndoPAT 2000 device (Itamar Medical Ltd. Caesarea, Israel) as previously described.(5) Finger probes were placed on symmetric fingers bilaterally, and a BP cuff was placed on one arm, with the other arm serving as control. PAT was continuously measured for 20 minutes. In between, for 5 minutes, BP cuff was inflated to supra systolic pressure in the test arm. At the end of the occlusion and dilatation periods, reactive hyperemia was captured as an increase in the PAT signal amplitude and compared with the control arm. A postocclusion to preocclusion ratio was calculated by EndoPAT software, providing RHI. Augmentation index (AI) was calculated from PAT pulses at the baseline period. The result was further normalized to heart rate of 75 bpm (AI@75), as previously described.(5)

3)SphygmoCor PWV and velocity system: Central aortic BP and stiffness were quantified using SphygmoCor CP system (AtCor Medical Pty Ltd.; New South Wales, Australia). The central aortic pressure PWV was determined by using the pressure tonometer and an EKG signal was used simultaneously to visualize ventricular-vascular interactions. Standard algorithm and procedures, as described elsewhere, were used to quantify results.(6)

Quantification of LDGs:

PBMCs were isolated by Ficoll-Paque density gradient and red blood cells were lysed with hypertonic solution. PBMCs were resuspended in 2% FBS/PBS, blocked for 15 minutes with Human TruStain Fc Receptor Blocking Solution (BioLegend; San Diego, CA), then resuspended in FACS buffer and incubated with fluorochrome-conjugated mouse anti–human-CD10 (clone H10A, catalog 312209), –CD15 (clone HI98, catalog 301906), and –CD14 (clone HCD14, catalog 325610) antibodies (BioLegend; San Diego, CA) or isotype controls for 15 minutes in the dark. Cells were fixed with 2% PFA. Data was collected using a BD FACSCanto RUO and analyzed using FlowJo Software Version 10. Cutoff values for positive staining were determined using compensation controls for each fluorophore. LDGs were classified as $CD10^{+}$, $CD15^{+}$, $CD14^{lo}$. (7)

NETs ELISA:

HNE-DNA NET complexes were measured in plasma, as described. (8) In brief, 96-well ELISA plates were coated overnight at 4°C with rabbit anti–human neutrophil elastase (HNE; Calbiochem; San Diego, CA). Plates were blocked in 1% BSA and incubated overnight with plasma in blocking buffer. After washing, plates were incubated for 1 hour at room temperature with anti-dsDNA (clone BV16-13, MilliporeSigma; Burlington, MA). Plates were washed and incubated for 1 hour with anti–mouse IgG-HRP conjugate (Bio-Rad), followed by a wash and the addition of TMB substrate and stop reagent (MilliporeSigma; Burlington, MA). Absorbance was measured at 450 nm, and values were calculated as an OD Index.

ELISAs for soluble markers of endothelial cell activation:

Serum sICAM-1, sVCAM-1, and sL-selectin levels were detected using commercially available ELISA kits (Invitrogen, BMS201, BMS232, BMS206) following manufacturer protocols.

Hasni S, *et al. Ann Rheum Dis* 2022; 81:1576–1584. doi: 10.1136/ard-2022-222658

^{18}F-FDG–PET/CT:

A subset of 30 subjects underwent this test, which was performed following an overnight fast. Images were obtained approximately 60 minutes after administration of 10 mCi of ^{18}F-FDG. All scans were completed using a 64-slice scanner (Siemens Biograph) acquiring 1.5 mm axial slices of the aorta. Standard bed positions of 3 minutes each were applied, and whole body scans were obtained for each patient from the vertex of the skull to the toes.. The extent of ^{18}F-FDG uptake within the aortic wall was measured with dedicated software (OsiriX MD, Pixmeo SARL). Each arterial region of interest produced 2 measures of metabolic activity: a mean standardized uptake value (SUV_{mean}) and a maximal SUV (SUV_{max}), which were obtained in the aorta from the aortic outflow tract to the abdominal aorta. Regions of interest were also placed on 10 contiguous slices over the superior vena cava to obtain a single average background blood activity. The SUV_{mean} from each of the superior vena cava slices were then averaged to produce 1 venous value. To account for background blood activity, SUV_{max} values from each aortic slice were divided by the average venous SUV_{mean} value to yield taeget/background ratio(TBR), a measure of vascular inflammation as previously described. (9)

RNA Isolation and IFN Gene Signature (ISG) quantification by Nanostring:

Total RNA was extracted from whole blood using Paxgene blood RNA isolation kit (PreAnalytiX, Switzerland). RNA concentration was measured by NanoDrop (Thermo Fisher Scientific). The nCounter Element prep kit (NanoString Technologies) was used for Nanostring assay. A NanoString TagSet consisting of fluorescently labeled specific Reporter Tags and a biotinylated universal Capture Tag were supplied by NanoString. There were 6 spike-in positive controls used to determine the hybridization efficiency, and 6 negative controls used to check non-specific background. A target-specific oligonucleotide probe pairs (synthesized by IDT, Coralville, IA) contained 37 ISGs, previously identified as discriminative of the IFN signature,

and 4 housekeeping genes (*ALAS1, HPRT1, TBP, TUBB*). A total of 100ng of RNA was used for hybridization at 67^0C for 16-21 hour on thermocycler. The hybridized samples were inserted into the nCounter Prep Station, where they were purified and immobilized onto the internal surface of a sample cartridge for 2-3 h. The sample cartridge was transferred to the nCounter Digital Analyzer where color codes were counted and tabulated for each target molecule. The resulting data were processed with nSolver software (NanoString Technologies), which included assessment of quality of the runs. Data were combined, normalized, and analyzed in Excel (Microsoft Corporation). Synthetic DNA oligonucleotides of each of the 37 ISGs and 4 housekeeping genes were used in each run as a calibration standard to check run and reagent lot consistency.

High dimensional transcriptional, and flow cytometry analysis.

Sample collection and selection for high dimensional analysis:

Peripheral blood was stored in PAXgene Blood RNA tubes for transcriptional analysis and viable PBMC were isolated and cryopreserved for flow cytometry according to Center for Human Immunology (CHI) protocols (https://chi.niaid.nih.gov/web/new/our-research/sop.html). A subgroup of 24 subjects were selected for high dimensional phenotyping, which comprised the 12 individuals from each study arm that demonstrated the greatest decreases in CAVI when treated with pioglitazone.

Transcriptomics:

RNA extraction was performed on a QIAsymphony SP instrument,

using QIAsymphony PAXgene Blood RNA Kits, with RNA yields determined on the Biotek Synergy H1 plate reader using Quant-IT Ribo Green Assay Kit, and RNA quality assessed on the Agilent 4200 TapeStation using RNA Screen Tape. RNA-seq libraries were prepared from 500 ng of total RNA on the Biomek FXp robot using Universal Plus mRNA-Seq kit with Human

Globin AnyDeplete. Library concentration was determined on the Biotek Synergy H1 plate reader using Quant-IT Pico Green dsDNA Assay Kit. Library size distribution was determined using D1000 ScreenTape on the 4200 TapeStation System. All libraries were diluted to the same molar concentration with a QIAgility liquid handling robot, equal volumes of normalized samples were pooled, and plate pools quantified by qPCR using KAPA Library Quantification Kit. One lane of a NovaSeq 6000 S4 run (200 cycles) was then used for sequencing at NHLBI Sequencing Core. Sequencing reads were adapter and quality trimmed then aligned to the human genome using STAR software, with read counts determined using HTSeqCount software, before normalization using the LIMMA packages and R software.

Flow Cytometry:

Cytometry used a Cytek Aurora with broad immune lineages assessed in unstimulated cells using a 27-color panel and manual quantification of 58 populations (**Supplemental Table 1A,B**). For more comprehensive characterization of T cell focused phenotypes a 34-color panel and manual quantification of 83 populations was performed (**Supplemental Table 2 A,B**), after in vitro stimulation with PMA (10 ng/mL) and ionomycin (500 ng/mL) in combination with protein transport inhibitors (Monensin 2uM, Brefeldin A 1 ug/mL) for 4 hours in a 37°C tissue culture incubator.

Data analysis:

Longitudinal changes in either gene expression or flow cytometry populations were evaluated by paired test, with *P* values corrected for multiple comparisons using the Benjamini-Hochberg method when described, using R-Shiny web tools developed in-house but similar to those previously described (10). For transcriptomic responses differentially expressed genes were used for gene set enrichment analyses performed using tmod and BTM that reflect biologically responding pathways (11, 12).

Statistical Analysis:

Sample size was calculated based on previous publications for arterial stiffness and vascular dysfunction in SLE and RA(5, 13-16). Based on our previous experience with the CV lupus cohort and on published literature on cross-over designs using PGZ to measure CV markers in other populations, dropout rate was estimated between 3-20%. Bring a proof of concept study, at least moderate differences in outcome were considered to be clinically meaningful. The actual analysis used data on all subjects including those who provided partial information. This sample was considered to provide power to detect moderate standardized differences for the other variables measured in this study.

Most of the outcome variables were measured at Day 1 (prior to randomization), month 3 (end of period 1), month 5 (start of period 2) and month 8 (end of period 2). Change score in these continuous outcomes was summarized by mean and standard deviation (SD): M3 – D1 for period 1 and M8 – M5 for period 2, for each sequence respectively. Linear mixed models were used to analyze the change scores in mean CAVI, PWI, and the log-transformed RHI. The models included the fixed effects of baseline value (D1 for period 1, M3 for period 2), treatment group (PGZ or placebo), period (1 or 2), and sequence (AB or BA). The random effect was "subject". Residual plots were used to assess model normality assumptions. For the treatment group difference (assessed by the difference in change scores between PGZ and placebo), the estimate, its associated confidence interval (CI), and p-values were reported from the mixed effects models. Other secondary efficacy endpoints were analyzed in a similar manner. The Wilcoxon rank sum test was used to analyze NETs when normality assumption was violated(43). For continuous variables only measured in period 1, analysis of covariance was used to analyze the data. Efficacy analyses were based on the intention to treat population, which includes all the subjects who were randomized. For the primary endpoints, 0.05/3 was set as the cutoff for statistical significance. For

Hasni S, *et al. Ann Rheum Dis* 2022; 81:1576–1584. doi: 10.1136/ard-2022-222658

all secondary efficacy endpoints, p-values and 95% confidence intervals were provided to examine the statistical evidence. All statistical analyses were performed using SAS (Version 9.4, SAS Institute, Cary, NC).

Supplemental Results

As the targeted analysis of inflammation-related genes showed no effect when subjects were treated with pioglitazone, we performed unbiased screening to detect potentially other effects of this drug on immune phenotype. For this we focused on analyzing a subgroup of 24 subjects, which comprised the 12 individuals from each study arm that demonstrated the greatest decreases in CAVI when treated with pioglitazone. Whole blood transcriptomic analysis was performed with changes between consecutive study timepoints used to rank genes for enrichment analyses of blood transcriptional modules (BTM). The number of significantly enriched pathways (FDR<0.05) was no higher in the study arms receiving pioglitazone than placebo for both of 0-3- and 5-8-month treatment periods (Supplemental Table 6A). Further, the pathways that did change with pioglitazone treatment showed no overlap between the two study arms (Supplemental Table 6 B, C). Next, PBMC were analyzed by high-dimensional flow cytometry, using a 27-color panel to quantify 58 populations spanning broad immune lineages in unstimulated cells, and a 34-color panel to quantify 83 populations that more comprehensively characterized T cell populations revealed by transcription factor or cytokine expression after in vitro stimulation with PMA. For both panels no significant changes in any population frequencies were observed between study timepoints after correction for multiple testing. The numbers of populations changing with nominal significance are reported (Supplemental Table 7 A, D), with those populations corresponding to the periods of drug treatment in each study arm also shown (Supplemental Table 7 B, C,E,F). Together this broad immune phenotyping analysis

indicated that although some longitudinal changes were observed in these 24 subjects, no changes in either peripheral blood transcriptional profiles or cell population frequencies could be attributed to treatment with pioglitazone.

Supplemental Figures:

Supplemental Figure 1

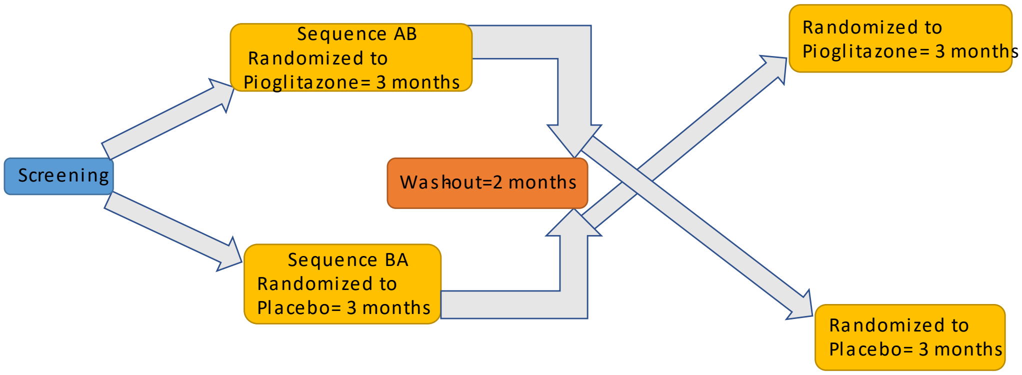

Supplemental Figure 1. Flow diagram of clinical trial. Subjects were randomized to either sequence AB (PGZ=3months, washout=2 months and placebo=3months) or sequence BA(placebo=3months, washout=2months and PGZ=3months).

Supplemental Figure 2

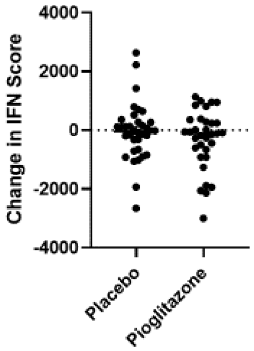

Supplemental Figure 2. Effect of pioglitazone on IFN gene signature. RNA was assessed for a ISGs by NanoString, generating an "IFN score" for each patient at each time point. Change in IFN score over the allocation period was not significantly different between patients receiving PGZ and patients receiving placebo.

Hasni S, *et al. Ann Rheum Dis* 2022; 81:1576–1584. doi: 10.1136/ard-2022-222658

Supplemental Figure 3

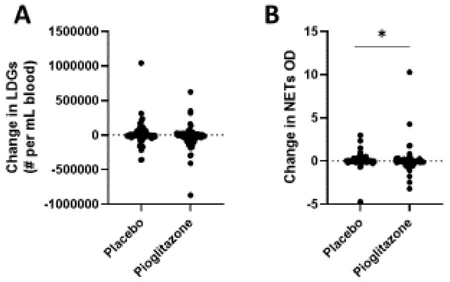

Supplemental Figure 3. Effect of PGZ on levels of circulating LDGs and NETs. **(A)** LDGs were quantified by flow cytometry for each patient before and after each allocation period. Change in levels of LDGs was not significantly different for patients when comparing the levels over the period on placebo to those over the period on PGZ. **(B)** Levels of NETs were assessed in plasma by quantifying HNE:DNA complexes. On PGZ, median level of NETs was lower compared to median level of NETs on placebo ($p = 0.026$).* = $p \leq 0.05$;

Supplemental Tables:

Supplemental Table 1. Twenty-seven-color panel for flow cytometry analysis of broad lineage populations in unstimulated PBMC.

1A) Antibody clones and fluors are detailed for the 27-color panel.

	**Specificity**	**Fluorochrome**	**Clone**	**vendor**	**cat#**
1	CD197	BUV395	150503	BD	CUSTOM
2	Dead cells	Live/Dead Blue		ThermoFisher	L23105
3	CD16	BUV496	3G8	BD	612944
4	HLA-DR	BUV661	G46-6	BD	612980
5	CD196	BUV737	11A9	BD	564377
6	CD183(CXCR3)	BUV805	IC6/CXCR3	BD	742048
7	IgD	BV421	IA6-2	BD	562518
8	CD4	eFluor450 (V450)	SK3	BD	560345
9	CD127	BV480	HIL-7R-M21	BD	566101
10	CD19	BV570	HIB19	BD	CUSTOM
11	CD194(CCR4)	BV605	1G1	Biolegend	359418
12	CD123	BV650	7G3	BD	563405
13	CD25	BV711	2A3	BD	563159
14	CD14	BV750	M5E2	BD	746920
15	CD27	BV786	L128	BD	563327
16	CD45RA	BB515	H100	BD	564552
17	CD38	PerCP-Cy5.5	HIT2	BD	551400
18	CD24	BB700	ML5	BD	566524
19	CD45	BB790	HI30	BD	CUSTOM
20	CD8	PE	RPA-T8	BD	555367
21	CD45RO	PE-Texa Red	UCHL1	Beckman Coulter	IM2712U
22	CD11c	PE-Cy5	B-Ly6	BD	551077
23	CD20	PE-Cy5.5	HI47	ThermoFisher	MHCD2018
24	CD185(CXCR5)	PE-Cy7	RF8B2	Biolegend	356924
25	CCR10	AlexaFluor 647 (APC)	314305	R&D Systems	FAB3478A
26	CD56	APC-R700	NCAM 16.2	BD	565139
27	CD3	APC-H7	SK7	BD	560176

Hasni S, *et al. Ann Rheum Dis* 2022; 81:1576–1584. doi: 10.1136/ard-2022-222658

1B) Panel of 58 subsets of PBMCs which were quantified are shown with the manual gates by which they were defined.

Pop #	Gate 1	Gate 2	Gate 3	Gate 4	Gate 5	Gate 6	Gate 7	Subset name I	Subset name II
1	singlet Live CD45+							Leukocytes	Leukocytes
2	singlet Live CD45+	CD3+CD19-						Total T cells	Total T cells
3	singlet Live CD45+	CD3+CD19-	CD4+CD8-					T helper cells (CD4+ T cells)	Th
4	singlet Live CD45+	CD3+CD19-	CD4+CD8-	CD38+				CD38+ activated T helper cells	CD38+ actTh
5	singlet Live CD45+	CD3+CD19-	CD4+CD8-	CD38+HLA-DR+				CD38+HLA-DR+ activated T helper cells	CD38+HLA-DR+actTh
6	singlet Live CD45+	CD3+CD19-	CD4+CD8-	CD45RA-				CD45RA- T helper cells	CD45RA-
7	singlet Live CD45+	CD3+CD19-	CD4+CD8-	CD45RA-	CXCR5+CCR10-			CXCR5+ T helper cells (T follicular helper)	Tfh
8	singlet Live CD45+	CD3+CD19-	CD4+CD8-	CD45RA-	CXCR5-			CXCR5- T helper cell subests	Th subsets
9	singlet Live CD45+	CD3+CD19-	CD4+CD8-	CD45RA-	CXCR5-	CCR6-		CXCR5-CCR6-T helper cell subsets	Th1_Th2_ThGM

Hasni S, *et al. Ann Rheum Dis* 2022; 81:1576–1584. doi: 10.1136/ard-2022-222658

10	singlet Live CD45+	CD3+CD19-	CD4+CD8-	CD45RA-	CXCR5-	CCR6-	CXCR3+CCR10-	CXCR3+CCR10-CCR6- T helper cells	Th1
11	singlet Live CD45+	CD3+CD19-	CD4+CD8-	CD45RA-	CXCR5-	CCR6-	CXCR3-CCR10-	CXCR3-CCR10-CCR6- T helper cells	Th2
12	singlet Live CD45+	CD3+CD19-	CD4+CD8-	CD45RA-	CXCR5-	CCR6-	CXCR3-CCR10+	CXCR3-CCR10+CCR6- T helper cells	Th_GM_CSF
13	singlet Live CD45+	CD3+CD19-	CD4+CD8-	CD45RA-	CXCR5-	CCR4-CCR6+		CCR4-CCR6+ T helper cells	Th9
14	singlet Live CD45+	CD3+CD19-	CD4+CD8-	CD45RA-	CXCR5-	CCR4+CCR6+		CCR4+CCR6+ T helper cells	Th22_Th17
15	singlet Live CD45+	CD3+CD19-	CD4+CD8-	CD45RA-	CXCR5-	CCR4+CCR6+	CCR10-	CCR4+CCR6+ CCR10-T helper cells	Th17
16	singlet Live CD45+	CD3+CD19-	CD4+CD8-	CD45RA-	CXCR5-	CCR4+CCR6+	CCR10+CXCR3-	CCR4+CCR6+CXCR3-CCR10+ T helper cells	Th22
17	singlet Live CD45+	CD3+CD19-	CD4+CD8-	CD127lowCD25high				CD127lowCD25high T helper cells	CD127lowCD25high_Th
18	singlet Live CD45+	CD3+CD19-	CD4+CD8-	CD127lowCD25high	CCR4+			T regulatory cells	Treg
19	singlet Live CD45+	CD3+CD19-	CD4+CD8-	CD127lowCD25high	CCR4+	HLA-DR+		Activated T regulatory cells (HLA-DR+)	actTreg

20	singlet Live CD45+	CD3+CD19-	CD4+CD8-	CD127lowCD25high	CCR4+	CD45RO+		Memory T regulatory cells	Memory Treg
21	singlet Live CD45+	CD3+CD19-	CD4+CD8-	CD127lowCD25high	CCR4+	CD45RO-		Naïve T regulatory cells	Naive Treg
22	singlet Live CD45+	CD3+CD19-	CD4+CD8-	CCR7+CD45RA-				Central memory T helper cells	CM_Th
23	singlet Live CD45+	CD3+CD19-	CD4+CD8-	CCR7-CD45RA+				Effector T helper cells	Eff_Th
24	singlet Live CD45+	CD3+CD19-	CD4+CD8-	CCR7-CD45RA-				Effector memory T helper cells	EM_Th
25	singlet Live CD45+	CD3+CD19-	CD4+CD8-	HLA-DR+				HLA-DR+ activated T helper cells	HLA-DR+actTh
26	singlet Live CD45+	CD3+CD19-	CD4+CD8-	CCR7+CD45RA+				Naïve T helper cells	Naive Th
27	singlet Live CD45+	CD3+CD19-	CD4-CD8+					Cytotoxic T cells (CD8+ T cells)	Tc
28	singlet Live CD45+	CD3+CD19-	CD4-CD8+	CD38+				CD38+ activated cytotoxic T cells	CD38+ actTc
29	singlet Live CD45+	CD3+CD19-	CD4-CD8+	CD38+HLA-DR+				CD38+ HLA-DR+ activated cytotoxic T cells	CD38+HLA-DR+Tc

30	singlet Live CD45+	CD3+CD19-	CD4-CD8+	CCR7+CD45RA-				Central memory cytotoxic T cells	CM Tc
31	singlet Live CD45+	CD3+CD19-	CD4-CD8+	CCR7-CD45RA+				Effector cytotoxic T cells	Eff Tc
32	singlet Live CD45+	CD3+CD19-	CD4-CD8+	CCR7-CD45RA-				Effector memory cytotoxic T cells	EM Tc
33	singlet Live CD45+	CD3+CD19-	CD4-CD8+	HLA-DR+				HLA-DR+ activated cytotoxic T cells	HLA-DR+actTc
34	singlet Live CD45+	CD3+CD19-	CD4-CD8+	CCR7+CD45RA+				Naïve cytotoxic T cells	Naive Tc
35	singlet Live CD45+	CD3+CD19-	CD4-CD8-					CD4-CD8- T cells	DNT
36	singlet Live CD45+	CD3+CD19-	CD4+CD8+					CD4+CD8+ T cells	DPT
37	singlet Live CD45+	CD3-CD19-						CD3-CD19- cells	CD3-CD19-
38	singlet Live CD45+	CD3-CD19-	CD14-HLA-DR+					Dendritic cells	DC
39	singlet Live CD45+	CD3-CD19-	CD14-HLA-DR+	CD11c+CD123-				Myeloid Dendritic cells	mDC

40	singlet Live CD45+	CD3-CD19-	CD14-HLA-DR+	CD11c-CD123+				Plasmacytoid Dendritic cells	pDC
41	singlet Live CD45+	CD3-CD19-	CD14-HLA-DR-					containing Granulocytes and NK cells	Gr_NK
42	singlet Live CD45+	CD3-CD19-	CD14-HLA-DR-	CD123+CD56-				Basophils	Basophils
43	singlet Live CD45+	CD3-CD19-	CD14-HLA-DR-	CD123-CD56-				Granulocytes	Gr
44	singlet Live CD45+	CD3-CD19-	CD14-HLA-DR-	CD123-CD56+				NK cells	NKs
45	singlet Live CD45+	CD3-CD19-	CD14-HLA-DR-	CD123-CD56+	CD16+			CD16+ NK cells	CD16+NKs
46	singlet Live CD45+	CD3-CD19-	CD14-HLA-DR-	CD123-CD56+	CD56highCD16low			CD56highCD16low NK cells	CD56highCD16low NKs
47	singlet Live CD45+	CD3-CD19-	CD14-HLA-DR-	CD123-CD56+	CD56lowCD16low			CD56lowCD16low NK cells	CD56lowCD16low NKs
48	singlet Live CD45+	CD3-CD19-	CD14+					Monocytes	Monocytes
49	singlet Live CD45+	CD3-CD19-	CD14+	CD16-				Classical Monocytes	Classical Mono

50	singlet Live CD45+	CD3-CD19-	CD14+	CD16-HLA-DR-				containing MDSCs subsets	containing MDSCs subsets
51	singlet Live CD45+	CD3-CD19-	CD14+	CD16+				Non-classical Monocytes	Non-classical Mono
52	singlet Live CD45+	CD3-CD19+						CD19+ B cells	CD19+ B cells
53	singlet Live CD45+	CD3-CD19+	CD20+					CD19+CD20+ transitional B cells	transitional B cells
54	singlet Live CD45+	CD3-CD19+	CD20+	CD24+CD38+				CD24+CD38+ transitional B cells	CD24+CD38+ transitional B
55	singlet Live CD45+	CD3-CD19+	CD20+	IgD-CD27-				IgD-CD27- B cells	IgD-CD27- B cells
56	singlet Live CD45+	CD3-CD19+	CD20+	IgD+CD27+				Memory IgD+ B cells	memory IgD+ B
57	singlet Live CD45+	CD3-CD19+	CD20+	IgD-CD27+				Memory IgD-B cells	memory IgD-B
58	singlet Live CD45+	CD3-CD19+	CD20+	IgD+CD27-				Naive B cells	Naive B cells

Supplemental Table 2. A 34-color panel for flow cytometry analysis of T cell focused populations in PMA-stimulated PBMC.

2A) Antibody clones and fluors are detailed for the 34 color panel.

	Target	Fluorochrome	clone	company	cat#
1	CD197 (CCR7)	BUV395	150503	BD	CUSTOM
2	CD26	BUV496	M-A261	BD	50667
3	Dead cells	Live/Dead Blue		ThermoFisher	L23105
4	CD161	BUV563	HP-3G10	BD	749223
5	CD25	BUV615	2A3	BD	612996
6	HLA-DR	BUV661	G46-6	BD	612980
7	CD56	BUV737	NCAM16.2	BD	612766
8	CD3	BUV805	UCHT1	BD	612895
9	IL-2	BV421	MQ1-17H12	BD	564164
10	Tbet	V450	O4-46	BD	561312
11	IFNg	BV480	B27	BD	566100
12	CD14	BV510 (Dump channel)	M5E2	BD	740163
	CD19	BV510 (Dump channel)	SJ25C1	BD	562947
	CD41a	BV510 (Dump channel)	HIP8	BD	563250
13	CD45	Pacific Orange	HI30	ThermoFisher	MHCD4530
14	TCRVa7.2	BV605	OF-5A12	BD	749491
15	CD4	Qdot 605	S3.5	ThermoFisher	Q10008
16	Ki67	BV650	B56	BD	563757
17	CD69	BV711	FN50	BD	563836
18	TNF	BV750	MAb11	BD	566359
19	CD196(CCR6)	BV785	G034E3	Biolegend	353422
20	CD8	Qdot 800	3B5	ThermoFisher	Q22157
21	IL-5	BB515	JES1-39D10	BD	CUSTOM
22	Granzyme B	FITC	GB11	Biolegend	515403
23	IL-13	BB660	JES10-5A2	BD	CUSTOM
24	CD45RA	PerCP-Cy5.5	HI100	BD	563429
25	GATA3	PerCP-eFluor710	TWAJ	ThermoFisher	46-9966-42
26	MR1	PE		NIH Tetramer Core Facility	hu MR1 5-OP-RU PE labeled tetramer
27	GM-CSF	PE/Dazzle 594	BVD2-21C11	Biolegend	502318
28	TCRgd	PE-Cy5	B1	BD	CUSTOM
29	FOXP3	PE-Cy5.5	PCH101	ThermoFisher	35-4776-42
30	IL-22	PE-Cy7	22URTI	ThermoFisher	25-7229-42
31	IL-4	APC	MP4-25D2	BD	554486
32	RORgt	Alexa Fluor 647	Q21-559	BD	563620
33	IL-17A	APC-R700	N49-653	BD	565163
34	Perforin	APC/Fire750	B-D48	BIolegend	353318

2B) A panel of 83 subsets of PBMCs which were quantified are shown with the manual gates by which they were defined.

**Pop #**	**Gate 1**	**Gate 2**	**Gate 3**	**Gate 4**	**Gate 5**	**Gate 6**	**Subset name**
1	singlet Live CD45+						Leukocytes(CD45+)
2	singlet Live CD45+	CD3+CD19-CD14-CD41a-					CD3+ T cells
3	singlet Live CD45+	CD3+CD19-CD14-CD41a-	CD161+ CD26+				CD161+CD26+ T cells
4	singlet Live CD45+	CD3+CD19-CD14-CD41a-	CD161+ CD26+	TCRValpha 7.2+MR1+			MAIT cells
5	singlet Live CD45+	CD3+CD19-CD14-CD41a-	CD3+CD56+				NKT cells
6	singlet Live CD45+	CD3+CD19-CD14-CD41a-	CD3+CD56+	GranzymeB+Perforin+			GranzymeB+Perforin+ NKT cells
7	singlet Live CD45+	CD3+CD19-CD14-CD41a-	CD3+CD56+	IFNg+			IFNg+ NKT cells
8	singlet Live CD45+	CD3+CD19-CD14-CD41a-	CD3+CD56+	IFNg+TNF+			IFNg+TNF+ NKT cells
9	singlet Live CD45+	CD3+CD19-CD14-CD41a-	CD3+CD56+	TNF+			TNF+ NKT cells
10	singlet Live CD45+	CD3+CD19-CD14-CD41a-	TCRgd-				T cells
11	singlet Live CD45+	CD3+CD19-CD14-CD41a-	TCRgd-	CD4+CD8-			T helper cells (Th)
12	singlet Live CD45+	CD3+CD19-CD14-CD41a-	TCRgd-	CD4+CD8-	IL-2+IFNg+		IL-2+IFNg+ Th cells
13	singlet Live CD45+	CD3+CD19-CD14-CD41a-	TCRgd-	CD4+CD8-	IL-2+TNF+		IL-2+TNF+ Th cells
14	singlet Live CD45+	CD3+CD19-CD14-CD41a-	TCRgd-	CD4+CD8-	CD25+Foxp3+		CD25+Foxp3+ Treg cells
15	singlet Live CD45+	CD3+CD19-CD14-CD41a-	TCRgd-	CD4+CD8-	CD25+Foxp3+	CD69+	Activated Foxp3+ Treg cells
16	singlet Live CD45+	CD3+CD19-CD14-CD41a-	TCRgd-	CD4+CD8-	CD69+		CD69+Th cells

Hasni S, *et al. Ann Rheum Dis* 2022; 81:1576–1584. doi: 10.1136/ard-2022-222658

17	singlet Live CD45+	CD3+CD19-CD14-CD41a-	TCRgd-	CD4+CD8-	CD69+Ki67+		CD69+Ki67+ Th cells
18	singlet Live CD45+	CD3+CD19-CD14-CD41a-	TCRgd-	CD4+CD8-	CD4+FoxP3+		Foxp3+ Treg cells
19	singlet Live CD45+	CD3+CD19-CD14-CD41a-	TCRgd-	CD4+CD8-	GATA3+IL-4+		GATA3+IL-4+ Th cells
20	singlet Live CD45+	CD3+CD19-CD14-CD41a-	TCRgd-	CD4+CD8-	GATA3+IL-5+		GATA3+IL-5+ Th cells
21	singlet Live CD45+	CD3+CD19-CD14-CD41a-	TCRgd-	CD4+CD8-	GATA3+IL-13+		GATA3+IL-13+ Th cells
22	singlet Live CD45+	CD3+CD19-CD14-CD41a-	TCRgd-	CD4+CD8-	GM-CSF+		GM-CSF+ Th cells
23	singlet Live CD45+	CD3+CD19-CD14-CD41a-	TCRgd-	CD4+CD8-	GM-CSF+IL-17A+		GM-CSF+IL-17A+ Th cells
24	singlet Live CD45+	CD3+CD19-CD14-CD41a-	TCRgd-	CD4+CD8-	IFNg+		IFNg+ Th cells
25	singlet Live CD45+	CD3+CD19-CD14-CD41a-	TCRgd-	CD4+CD8-	IFNg+TNF+		IFNg+TNF+ Th cells
26	singlet Live CD45+	CD3+CD19-CD14-CD41a-	TCRgd-	CD4+CD8-	IL-2+		IL-2+ Th cells
27	singlet Live CD45+	CD3+CD19-CD14-CD41a-	TCRgd-	CD4+CD8-	IL-4+		IL-4+ Th cells
28	singlet Live CD45+	CD3+CD19-CD14-CD41a-	TCRgd-	CD4+CD8-	IL-4+IL-5+		IL-4+IL-5+ Th cells
29	singlet Live CD45+	CD3+CD19-CD14-CD41a-	TCRgd-	CD4+CD8-	IL-4+IL-13+		IL-4+IL-13+ Th cells
30	singlet Live CD45+	CD3+CD19-CD14-CD41a-	TCRgd-	CD4+CD8-	IL-5+		IL-5+ Th cells
31	singlet Live CD45+	CD3+CD19-CD14-CD41a-	TCRgd-	CD4+CD8-	IL-13+		IL-13+ Th cells
32	singlet Live CD45+	CD3+CD19-CD14-CD41a-	TCRgd-	CD4+CD8-	IL-13+IL-5+		IL-13+IL-5+ Th cells
33	singlet Live CD45+	CD3+CD19-CD14-CD41a-	TCRgd-	CD4+CD8-	IL-17A+		IL-17A+ Th cells
34	singlet Live CD45+	CD3+CD19-CD14-CD41a-	TCRgd-	CD4+CD8-	IL-17A+IL-22+		IL-17A+IL-22+ Th cells

Hasni S, *et al. Ann Rheum Dis* 2022; 81:1576–1584. doi: 10.1136/ard-2022-222658

35	singlet Live CD45+	CD3+CD19-CD14-CD41a-	TCRgd-	CD4+CD8-	IL-22+		IL-22+ Th cells
36	singlet Live CD45+	CD3+CD19-CD14-CD41a-	TCRgd-	CD4+CD8-	IL-22+GM-CSF+		IL-22+GM-CSF+ Th cells
37	singlet Live CD45+	CD3+CD19-CD14-CD41a-	TCRgd-	CD4+CD8-	Ki67+		Ki67+ Th cells
38	singlet Live CD45+	CD3+CD19-CD14-CD41a-	TCRgd-	CD4+CD8-	RORgT+		RORgT+ Th cells
39	singlet Live CD45+	CD3+CD19-CD14-CD41a-	TCRgd-	CD4+CD8-	RORgT+IL-17A+		RORgT+IL-17A+ Th cells
40	singlet Live CD45+	CD3+CD19-CD14-CD41a-	TCRgd-	CD4+CD8-	Tbet+IFNg+		Tbet+IFNg+ Th cells
41	singlet Live CD45+	CD3+CD19-CD14-CD41a-	TCRgd-	CD4+CD8-	Tbet+IL-2+		Tbet+IL-2+ Th cells
42	singlet Live CD45+	CD3+CD19-CD14-CD41a-	TCRgd-	CD4+CD8-	Tbet+TNF+		Tbet+TNF+ Th cells
43	singlet Live CD45+	CD3+CD19-CD14-CD41a-	TCRgd-	CD4+CD8-	TNF+		TNF+ Th cells
44	singlet Live CD45+	CD3+CD19-CD14-CD41a-	TCRgd-	CD4+CD8-	CCR7+CD45RA-		Central Memory Th cells (CM Th)
45	singlet Live CD45+	CD3+CD19-CD14-CD41a-	TCRgd-	CD4+CD8-	CCR7-CD45RA+		Effector Th cells (Eff Th)
46	singlet Live CD45+	CD3+CD19-CD14-CD41a-	TCRgd-	CD4+CD8-	CCR7-CD45RA-		Effector Memory Th cells (EM Th)
47	singlet Live CD45+	CD3+CD19-CD14-CD41a-	TCRgd-	CD4+CD8-	GATA3+		GATA3+Th2 cells
48	singlet Live CD45+	CD3+CD19-CD14-CD41a-	TCRgd-	CD4+CD8-	CCR7+CD45RA+		Naive Th cells
49	singlet Live CD45+	CD3+CD19-CD14-CD41a-	TCRgd-	CD4+CD8-	CD25-Foxp3-		non-Treg cells
50	singlet Live CD45+	CD3+CD19-CD14-CD41a-	TCRgd-	CD4+CD8-	RORgT+IL-22+		RORgT+IL-22+ Th cells
51	singlet Live CD45+	CD3+CD19-CD14-CD41a-	TCRgd-	CD4+CD8-	Tbet+		Tbet+ Th1 cells
52	singlet Live CD45+	CD3+CD19-CD14-CD41a-	TCRgd-	CD4-CD8+			Cytotoxic T cell (Tc)

Hasni S, *et al. Ann Rheum Dis* 2022; 81:1576–1584. doi: 10.1136/ard-2022-222658

53	singlet Live CD45+	CD3+CD19-CD14-CD41a-	TCRgd-	CD4-CD8+	CD69+		CD69+Tc
54	singlet Live CD45+	CD3+CD19-CD14-CD41a-	TCRgd-	CD4-CD8+	Granzyme B+		GranzymeB+Tc
55	singlet Live CD45+	CD3+CD19-CD14-CD41a-	TCRgd-	CD4-CD8+	IFNg+		IFNg+Tc
56	singlet Live CD45+	CD3+CD19-CD14-CD41a-	TCRgd-	CD4-CD8+	IFNg+IL-2+		IFNg+IL-2+ Tc
57	singlet Live CD45+	CD3+CD19-CD14-CD41a-	TCRgd-	CD4-CD8+	IFNg+TNF+		IFNg+TNF+ Tc
58	singlet Live CD45+	CD3+CD19-CD14-CD41a-	TCRgd-	CD4-CD8+	IL-2+		IL-2+Tc
59	singlet Live CD45+	CD3+CD19-CD14-CD41a-	TCRgd-	CD4-CD8+	IL-2+TNF+		IL-2+TNF+Tc
60	singlet Live CD45+	CD3+CD19-CD14-CD41a-	TCRgd-	CD4-CD8+	Ki67+		Ki67+Tc
61	singlet Live CD45+	CD3+CD19-CD14-CD41a-	TCRgd-	CD4-CD8+	Ki67+CD69+		Ki67+CD69+Tc
62	singlet Live CD45+	CD3+CD19-CD14-CD41a-	TCRgd-	CD4-CD8+	Perforin+		Perforin+ Tc
63	singlet Live CD45+	CD3+CD19-CD14-CD41a-	TCRgd-	CD4-CD8+	Perforin+GranzymeB+		Perforin+GranzymeB+Tc
64	singlet Live CD45+	CD3+CD19-CD14-CD41a-	TCRgd-	CD4-CD8+	TNF+		TNF+ Tc
65	singlet Live CD45+	CD3+CD19-CD14-CD41a-	TCRgd-	CD4-CD8+	CCR7+CD45RA-		Central Memory Tc (CM Tc)
66	singlet Live CD45+	CD3+CD19-CD14-CD41a-	TCRgd-	CD4-CD8+	CCR7-CD45RA+		Effector Tc (Eff Tc)
67	singlet Live CD45+	CD3+CD19-CD14-CD41a-	TCRgd-	CD4-CD8+	CCR7-CD45RA-		Effector Memory Tc (EM Tc)
68	singlet Live CD45+	CD3+CD19-CD14-CD41a-	TCRgd-	CD4-CD8+	CCR7+CD45RA+		Naive Tc
69	singlet Live CD45+	CD3+CD19-CD14-CD41a-	TCRgd+				gd T cells (Tgd)
70	singlet Live CD45+	CD3+CD19-CD14-CD41a-	TCRgd+	GranzymeB+			GranzymeB+ gd T cells

71	singlet Live CD45+	CD3+CD19-CD14-CD41a-	TCRgd+	IFNg+			IFNg+ gd T cells
72	singlet Live CD45+	CD3+CD19-CD14-CD41a-	TCRgd+	IFNg+Perforin+			IFNg+Perforin+ gdT cells
73	singlet Live CD45+	CD3+CD19-CD14-CD41a-	TCRgd+	IFNg+TNF+			IFNg+TNF+ gdT cells
74	singlet Live CD45+	CD3+CD19-CD14-CD41a-	TCRgd+	Perforin+			Perforin+ gdT cells
75	singlet Live CD45+	CD3+CD19-CD14-CD41a-	TCRgd+	Perforin+GranzymeB+			Perforin+GranzymeB+ gdT cells
76	singlet Live CD45+	CD3+CD19-CD14-CD41a-	TCRgd+	TNF+			TNF+ gdT cells
77	singlet Live CD45+	CD3+CD19-CD14-CD41a-	TCRgd+	TNF+Perforin+			TNF+Perforin+ gdT cells
78	singlet Live CD45+	CD3-CD19-CD14-CD41a-					CD3-CD19-CD14-CD41a-
79	singlet Live CD45+	CD3-CD19-CD14-CD41a-	CD3-CD56+				CD56+ NK cells
80	singlet Live CD45+	CD3-CD19-CD14-CD41a-	CD3-CD56+	CCR6+CD69+			CCR6+CD69+ NK cells
81	singlet Live CD45+	CD3-CD19-CD14-CD41a-	CD3-CD56+	CD69+TNF+			CD69+TNF+ NK cells
82	singlet Live CD45+	CD3-CD19-CD14-CD41a-	CD3-CD56+	IFNg+CD69+			IFNg+CD69+ NK cells
83	singlet Live CD45+	CD3-CD19-CD14-CD41a-	CD3-CD56+	Perforin+GranzymeB+			Perforin+GranzymeB+ NK cells

Hasni S, *et al. Ann Rheum Dis* 2022; 81:1576–1584. doi: 10.1136/ard-2022-222658

Supplemental Table 3.

SLE disease activity and patient reported outcomes by sequence and period

	Sequence AB (pioglitazone/placebo) N=39				Sequence BA (placebo/pioglitazone) N=41					
	Period1 (pioglitazone)		Period2 (placebo)		Period1 (placebo)		Period2 (pioglitazone)		Treatment Effect*	
Variable (Mean±SD)	Baseline	Change	Baseline	Change	Baseline	Change	Baseline	Change	Estimate (95%CI)	P-value
SLEDAI-2K	5.13±2.75	-0.78±1.46	4.06±2.14	0.22±1.99	5.07±3.04	-1.03±2.66	3.58±2.79	-0.72±2.11	-0.42 (-1.00, 0.16)	0.15
C3 mg/dL	98.53±23.8	-1.01±13.08	99.04±23.73	2.77±9.88	105.65±25.9	0.76±13.44	107.36±22.64	-0.84±12.86	-2.55 (-6.26, 1.17)	0.18
C4 mg/dL	17.19±7.49	0.17±4.74	16.79±7.13	0.72±2.69	19.22±10.08	0.54±3.16	20.17±9.53	-1.38±3.66	-1.22 (-2.37, -0.07)	0.04
SF36 Total	111.69±9.13	0.94±7.29	111.47±8.23	-0.67±6.47	112.12±7.88	-0.2±6.43	112.61±7.6	1.33±6.54	1.59 (-0.21, 3.38)	0.08
PGA	0.42±0.2	-0.06±0.18	0.32±0.12	0.02±0.26	0.38±0.21	-0.01±0.25	0.32±0.18	-0.06±0.15	-0.05 (-0.12, 0.01)	0.12

Anti-dsDNA data analysis results: 59 subjects had their anti-dsDNA status unchanged (stayed negative or positive) in both periods, 7 showed improvement (DNA status changed from positive to negative) under PGZ and 3 showed improvement under placebo. All other subjects had mixed results.

Data are mean±SD. Change is defined as the post baseline value minus the baseline value during the period: i.e., M3 – D1 for period 1, M8 – M5 for period 2.
SLEDAI 2K = Systemic Lupus Erythematosus Disease Activity Index 2000; Anti-ds-DNA IU/mL= Anti double-stranded DNA antibody international unit/milliliter; C3 mg/dL= Complement protein C3 milligram/deciliter; C4 mg/dL= Complement protein C4 milligram/deciliter; SF36= Short Form 36; PGA= Physician global assessment

*: Linear mixed effects models were used to calculate the estimated treatment effect (the treatment group difference in the change score between pioglitazone and the placebo), its 95% CI, and the p-value.

Hasni S, *et al. Ann Rheum Dis* 2022; 81:1576–1584. doi: 10.1136/ard-2022-222658

Supplemental Table 4. Adverse events by severity

**Body System Preferred Term**	**Adverse Event Severity**				
	Mild n (%)	Moderate n (%)	Severe n (%)	Life-Threatening n (%)	Overall n (%)
**Blood and lymphatic system disorders**	3 (1.2)	3 (1.2)	0 (0)	0 (0)	6 (2.4)
**Cardiac disorders**	5 (2.01)	0 (0)	0 (0)	0 (0)	5 (2.0)
**Eye disorders**	3 (1.2)	1 (0.4)	0 (0)	0 (0)	4 (1.6)
**Gastrointestinal disorders**	26 (10.4)	6 (2.4)	1 (0.4)	0 (0)	33 (13.3)
**General disorders**	15 (6.0)	2 (0.8)	0 (0)	0 (0)	17 (6.8)
**Immune system disorders**	1 (0.4)	0 (0)	0 (0)	0 (0)	1 (0.4)
**Infections and infestations**	19 (7.6)	28 (11.2)	5 (2.0)	1 (0.4)	53 (21.3)
**Injury poisoning and procedural complications**	0 (0)	1 (0.4)	0 (0)	0 (0)	1 (0.4)
**Investigations**	22 (8.8)	11 (4.4)	4 (1.6)	0 (0)	37 (14.9)
**Metabolism and nutrition disorders**	7 (2.8)	1 (0.4)	0 (0)	0 (0)	8 (3.2)
**Musculoskeletal and connective tissue disorders**	5 (2.0)	4 (1.6)	0 (0)	0 (0)	9 (3.6)
**Nervous system disorders**	31 (12.5)	3 (1.2)	0 (0)	0 (0)	34 (13.7)
**Psychiatric disorders**	4 (1.6)	0 (0)	0 (0)	0 (0)	4 (1.6)
**Renal and urinary disorders**	7 (2.81)	0 (0)	0 (0)	0 (0)	7 (2.8)
**Reproductive system and breast disorders**	2 (0.8)	0 (0)	0 (0)	0 (0)	2 (0.8)
**Respiratory thoracic and mediastinal disorders**	12 (4.8)	7 (2.8)	0 (0)	0 (0)	19 (7.6)
**Skin and subcutaneous tissue disorders**	4 (1.6)	1 (0.4)	0 (0)	0 (0)	5 (2.0)
**Surgical and medical procedures**	0 (0)	0 (0)	1 (0.4)	0 (0)	1 (0.4)
**Vascular disorders**	2 (0.8)	0 (0)	1 (0.4)	0 (0)	3 (1.2)
**Total**	168 (67.5)	68 (27.3)	12 (4.82)	1 (0.4)	249 (100)

Hasni S, *et al. Ann Rheum Dis* 2022; 81:1576–1584. doi: 10.1136/ard-2022-222658

Supplemental Table 5. Summary of safety laboratory values by sequence and period

	Sequence AB (pioglitazone/placebo) N=39				Sequence BA (placebo/pioglitazone) N=41					
	Period1(pioglitazone)		Period2(placebo)		Period1(placebo)		Period2(pioglitazone)		Treatment Effect*	
Variable (Mean+SD)	Baseline	Change	Baseline	Change	Baseline	Change	Baseline	Change	Estimate (95%CI)	P-value
AST u/L	22.13±7.98	0.28±4.74	24.61±16.92	-2.31±10.11	24.88±13.71	-1.05±13.15	26.42±13.22	-2.19±13.52	0.75(-2.39,3.9)	0.64
ALT u/L	18.56±8.31	-0.72±8.09	22.42±22.51	-3.08±13.9	22.2±18.4	-0.5±16.14	26.34±24.53	-6.86±23.3	-1.58(-6.01,2.84)	0.48
CRP mg/L	3.46±5.07	-1.84±4.31	2.66±5.12	0.35±5.85	4.35±6.38	-0.94±7.08	2.67±2.61	1.16±9.5	-0.04(-2.31,2.23)	0.97
ESR mm/hr	24.26±22.55	-2.33±10.25	20.61±21.35	1.56±7.73	20.76±14.7	-1.43±10.3	20.37±15.11	1.92±10.8	-0.28(-3.29,2.74)	0.85
WBC K/mcL	5.11±2.04	-0.75±1.33	5.27±2.12	-0.27±1.51	5.02±1.33	-0.03±1.49	4.91±1.29	-0.72±1.29	-0.58(-1.03,-0.14)	0.01
RBC M/uL	4.32±0.58	-0.21±0.26	4.29±0.55	0.05±0.23	4.3±0.49	0.02±0.28	4.31±0.47	-0.19±0.25	-0.24(-0.32,-0.15)	<.0001
Hemoglobin g/dL	12.66±1.48	-0.46±0.94	12.71±1.36	0.04±0.8	12.51±1.51	-0.02±0.76	12.49±1.43	-0.47±0.68	-0.48(-0.74,-0.22)	0.0004
Hematocrit %	38.74±3.98	-1.2±2.58	38.7±3.4	0.24±2.19	38.07±4.15	0.03±2.33	38.22±3.76	-1.26±2.36	-1.36(-2.11,-0.61)	0.0005
Platelet K/mcL	234.74±67.97	-22.42±35.34	220.97±52.42	11.36±30.39	225.9±62.22	7.95±50.46	223.53±63.1	-18.19±29.98	-30.1(-42.32,-17.89)	<.0001
BUN mg/dL	13.18±4.89	2±3.49	13.33±5.13	-0.58±3.47	12.59±3.54	1.05±3.21	13.11±4.78	1.75±4.46	1.64(0.51,2.77)	0.005
Creatinine mg/dL	0.74±0.14	0.04±0.07	0.73±0.15	0.01±0.08	0.74±0.2	0.01±0.06	0.73±0.17	0.03±0.07	0.02(0.002,0.05)	0.03

Data are mean±SD. Change is defined as the post baseline value minus the baseline value during the period: i.e., M3 – D1 for period 1, M8 – M5 for period 2.
AST u/L= Aspartate aminotransferase units/liter; ALT u/L = Alanine aminotransferase units/liter; CRP mg/L = C-Reactive protein milligram/liter; ESR mm/hr = Erythrocyte sedimentation rate millimeters/hour; WBC K/mcL = White blood cell count thousands/cubic milliliter; RBC M/uL = Red blood cells count million/microliter; g/dL= grams/deciliter; BUN mg/dL= Blood urea nitrogen; milligrams/deciliter

*: Linear mixed effects models were used to calculate the estimated treatment effect (the treatment group difference in the change score between pioglitazone and the placebo), its 95% CI, and the p-value.

Hasni S, *et al. Ann Rheum Dis* 2022; 81:1576–1584. doi: 10.1136/ard-2022-222658

Supplemental Table 6. Longitudinal transcriptomic profiling of whole blood from 24 subjects.

6A) For 12 subjects from each study arm, comparing between consecutive study timepoints, changes in expression of all genes were used for enrichment analyses of BTM. The number of significantly enriched pathways are reported (FDR<0.05) and periods of pioglitazone treatment highlighted in red.

	**0 vs 3 months**	**3 vs 5 months**	**5 vs 8 months**
**AB arm**	44	30	10
**BA arm**	69	28	7

6B) In the AB arm gene expression changes spanning the pioglitazone treatment period from months 0 to 3 were enriched for 44 pathways shown.

**Pathway name**	**padj**	**NES**
platelet activation (I) (M32.0)	0.004531	2.386684
platelet activation (II) (M32.1)	0.004531	2.570327
CORO1A-DEF6 network (I) (M32.2)	0.004531	2.227201
CORO1A-DEF6 network (II) (M32.4)	0.004531	2.193038
cytoskeletal remodeling (M32.8)	0.004531	2.416415
BCR signaling (M54)	0.004531	2.242604
regulation of localization (GO) (M63)	0.004531	2.375884
enriched in cell cycle (M167)	0.004531	2.461214
chaperonin mediated protein folding (I) (M204.0)	0.004531	-2.38958
proteasome (M226)	0.004531	-2.39559
translation initiation (M227)	0.004531	-2.27608

spliceosome (M250)	0.004531	-2.41079
transcription regulation in cell development (M49)	0.004535	2.214802
enriched in T cells (I) (M7.0)	0.005438	-1.89505
enriched in NK cells (I) (M7.2)	0.005438	-1.94443
mismatch repair (I) (M22.0)	0.005438	-1.99312
KLF12 targets network (M32.3)	0.005438	2.126701
MAPK, RAS signaling (M100)	0.005438	2.000217
cell cycle, ATP binding (M144)	0.005438	-2.12247
mitosis (TF motif CCAATNNSNNNGCG) (M169)	0.005438	-2.21195
heme biosynthesis (I) (M171)	0.005438	2.127342
erythrocyte differentiation (M173)	0.005438	1.975573
chaperonin mediated protein folding (II) (M204.1)	0.005438	-2.20546
respiratory electron transport chain (mitochondrion) (M216)	0.005438	-2.10727
respiratory electron transport chain (mitochondrion) (M219)	0.005438	-2.36719
respiratory electron transport chain (mitochondrion) (M238)	0.005438	-2.10437
intracellular transport (M147)	0.006423	-2.09795
respiratory electron transport chain (mitochondrion) (M231)	0.006423	-2.02438
enriched for ubiquitination (M138)	0.015695	-1.9272

nuclear pore, transport; mRNA splicing, processing (M143)	0.021452	-1.89463
enriched in membrane proteins (M124)	0.022968	1.895463
regulation of transcription, transcription factors (M213)	0.024864	-1.93951
golgi membrane (II) (M237)	0.024864	-1.93583
enriched for TF motif PAX3 (M179)	0.025604	-1.86712
mitochondrial cluster (M235)	0.025858	-1.88417
nuclear pore complex (M106.0)	0.031756	-1.82812
phosphatidylinositol signaling system (M101)	0.032571	-1.83433
T cell activation (I) (M7.1)	0.035241	-1.68189
heme biosynthesis (II) (M222)	0.035241	1.807254
enriched in T cells (II) (M223)	0.035241	-1.82317
Resting dendritic cell surface signature (S10)	0.03856	1.634133
cell cycle, mitotic phase (M230)	0.03897	-1.80618
translation initiation factor 3 complex (M245)	0.043798	-1.77373
CD4 T cell surface signature Th2-stimulated (S7)	0.043798	-1.78455

6C) In the BA gene expression changes spanning the pioglitazone treatment period from months 5 to 8 were enriched for 7 pathways shown. For each pathway adjusted p values (padj) and normalized enrichment scores (NES) are reported.

Pathway name	padj	NES
enriched in monocytes (II) (M11.0)	0.016859	1.777754
intracellular transport (M147)	0.016859	2.060838
DC surface signature (S5)	0.016859	2.121322
cell cycle, ATP binding (M144)	0.019504	1.994704
cell division - E2F transcription network (M4.8)	0.025238	1.974083
platelet activation (II) (M32.1)	0.037629	-1.84243
endoplasmic reticulum (M37.2)	0.037629	1.906045

Hasni S, *et al. Ann Rheum Dis* 2022; 81:1576–1584. doi: 10.1136/ard-2022-222658

Supplemental Table 7. Longitudinal high-dimensional cytometry phenotyping of 24 subjects.

7A) Broad immune lineages were assessed in unstimulated PBMC using a 27-color panel quantifying 58 populations. For 12 subjects from each study arm, comparing between consecutive study timepoints, changes in frequency for each population were assessed by paired t test. The number of populations changing with nominal significance are reported ($p<0.05$) with periods of pioglitazone treatment highlighted in red.

	**0 vs 3 months**	**3 vs 5 months**	**5 vs 8 months**
**AB arm**	6	4	1
**BA arm**	0	3	4

7B) In the AB arm 6 populations which showed nominally significant changes between months 0 and 3 when treated with pioglitazone are reported.

**Population name**	**p value**	**p adj**	**change**
IgD-CD27- B cells	0.003499328	0.199461694	up
CD38+ HLA-DR+ activated cytotoxic T cells	0.013402901	0.311747941	up
CXCR3+CCR10-CCR6- T helper cells (Th1)	0.018423396	0.311747941	down
CD38+ activated cytotoxic T cells (actTc)	0.021877048	0.311747941	up
CXCR3-CCR10-CCR6- T helper cells (Th2)	0.031399249	0.357951437	up
Memory IgD+ B cells	0.047990365	0.455908466	down

7C) In the BA arm 4 populations which showed nominally significant changes between months 5 and 8 when treated with pioglitazone are reported.

**Population name**	**p value**	**p adj**	**change**
Central memory T helper cells	0.003078447	0.175471491	down
CCR4+CCR6+ T helper cells (TH22_Th17)	0.008299668	0.236540551	down
Myeloid Dendritic cells	0.02680646	0.457905801	up
CXCR5-CCR6-T helper cell subsets (Th1_Th2_ThGM-CSF)	0.03213374	0.457905801	down

7D) A more comprehensive analysis of T cell phenotypes was assessed in PBMC stimulated with PMA using a 34-color panel quantifying 83 populations. Again for 12 subjects from each study arm, comparing between consecutive study timepoints, changes in frequency for each population were assessed by paired t test and the number of populations changing with nominal significance are reported ($p<0.05$), with periods of pioglitazone treatment highlighted in red.

	**0 vs 3 months**	**3 vs 5 months**	**5 vs 8 months**
**AB arm**	2	12	2
**BA arm**	5	7	8

Hasni S, *et al. Ann Rheum Dis* 2022; 81:1576–1584. doi: 10.1136/ard-2022-222658

7E) In the AB arm 2 populations which showed nominally significant changes between months 0 and 3 when treated with pioglitazone are reported.

**Population name**	**p value**	**p adj**	**change**
IL-17A+IL-22+ Th cells	0.029328693	0.871776657	down
RORgT+IL-22+ Th cells	0.040843106	0.871776657	down

7F) In the BA arm 8 populations which showed nominally significant changes between months 5 and 8 when treated with pioglitazone are reported.

**Population name**	**p value**	**p adj**	**change**
GATA3+Th2 cells	0.003899897	0.283905385	down
Tbet+ Th1 cells	0.00959779	0.283905385	up
Tbet+TNF+ Th cells	0.015756715	0.283905385	up
Naive Tc	0.016004047	0.283905385	down
Perforin+ Tc	0.019028108	0.283905385	up
Perforin+GranzymeB+Tc	0.020773565	0.283905385	up
Effector Tc	0.038717688	0.435032839	up
GranzymeB+Tc	0.049714765	0.435032839	up

References:

1. Matthews DR, Hosker JP, Rudenski AS, Naylor BA, Treacher DF, Turner RC. Homeostasis model assessment: insulin resistance and beta-cell function from fasting plasma glucose and insulin concentrations in man. Diabetologia. 1985;28(7):412-9.
2. Mehta NN, Li R, Krishnamoorthy P, Yu Y, Farver W, Rodrigues A, et al. Abnormal lipoprotein particles and cholesterol efflux capacity in patients with psoriasis. Atherosclerosis. 2012;224(1):218-21.
3. Carlucci PM, Purmalek MM, Dey AK, Temesgen-Oyelakin Y, Sakhardande S, Joshi AA, et al. Neutrophil subsets and their gene signature associate with vascular inflammation and coronary atherosclerosis in lupus. JCI Insight. 2018;3(8).
4. Ichihara A, Yamashita N, Takemitsu T, Kaneshiro Y, Sakoda M, Kurauchi-Mito A, et al. Cardio-ankle vascular index and ankle pulse wave velocity as a marker of arterial fibrosis in kidney failure treated by hemodialysis. Am J Kidney Dis. 2008;52(5):947-55.
5. Marder W, Khalatbari S, Myles JD, Hench R, Lustig S, Yalavarthi S, et al. The peroxisome proliferator activated receptor-γ pioglitazone improves vascular function and decreases disease activity in patients with rheumatoid arthritis. J Am Heart Assoc. 2013;2(6):e000441.
6. Doupis J, Papanas N, Cohen A, McFarlan L, Horton E. Pulse Wave Analysis by Applanation Tonometry for the Measurement of Arterial Stiffness. Open Cardiovasc Med J. 2016;10:188-95.
7. Denny MF, Yalavarthi S, Zhao W, Thacker SG, Anderson M, Sandy AR, et al. A distinct subset of proinflammatory neutrophils isolated from patients with systemic lupus erythematosus induces vascular damage and synthesizes type I IFNs. J Immunol. 2010;184(6):3284-97.
8. Lood C, Blanco LP, Purmalek MM, Carmona-Rivera C, De Ravin SS, Smith CK, et al. Neutrophil extracellular traps enriched in oxidized mitochondrial DNA are interferogenic and contribute to lupus-like disease. Nat Med. 2016;22(2):146-53.
9. Naik HB, Natarajan B, Stansky E, Ahlman MA, Teague H, Salahuddin T, et al. Severity of Psoriasis Associates With Aortic Vascular Inflammation Detected by FDG PET/CT and Neutrophil Activation in a Prospective Observational Study. Arterioscler Thromb Vasc Biol. 2015;35(12):2667-76.
10. Cheung F, Fantoni G, Conner M, Sellers BA, Kotliarov Y, Candia J, et al. Web Tool for Navigating and Plotting SomaLogic ADAT Files. J Open Res Softw. 2017;5.
11. Subramanian A, Tamayo P, Mootha VK, Mukherjee S, Ebert BL, Gillette MA, et al. Gene set enrichment analysis: a knowledge-based approach for interpreting genome-wide expression profiles. Proc Natl Acad Sci U S A. 2005;102(43):15545-50.
12. Li S, Rouphael N, Duraisingham S, Romero-Steiner S, Presnell S, Davis C, et al. Molecular signatures of antibody responses derived from a systems biology study of five human vaccines. Nat Immunol. 2014;15(2):195-204.
13. Juárez-Rojas JG, Medina-Urrutia AX, Jorge-Galarza E, Caracas-Portilla NA, Posadas-Sánchez R, Cardoso-Saldaña GC, et al. Pioglitazone improves the cardiovascular profile in patients with uncomplicated systemic lupus erythematosus: a double-blind randomized clinical trial. Lupus. 2012;21(1):27-35.
14. Selzer F, Sutton-Tyrrell K, Fitzgerald S, Tracy R, Kuller L, Manzi S. Vascular stiffness in women with systemic lupus erythematosus. Hypertension. 2001;37(4):1075-82.
15. Bjarnegråd N, Bengtsson C, Brodszki J, Sturfelt G, Nived O, Länne T. Increased aortic pulse wave velocity in middle aged women with systemic lupus erythematosus. Lupus. 2006;15(10):644-50.
16. Brodszki J, Bengtsson C, Länne T, Nived O, Sturfelt G, Marsál K. Abnormal mechanical properties of larger arteries in postmenopausal women with systemic lupus erythematosus. Lupus. 2004;13(12):917-23.
